# Supplementary material for: Helicity dependent photocurrent in electrically gated (Bi1−xSbx)2Te3 thin films
Source: Nat Commun. 2017 Oct 19;8:1037. doi: 10.1038/s41467-017-00711-4 (PMC5648839; doi:10.1038/s41467-017-00711-4)
Supplement: Supplementary file 1 — Supplementary Figures [file 41467_2017_711_MOESM1_ESM.pdf]

File Name: Supplementary Information
Descriptions: Supplementary Figures, Supplementary Notes and Supplementary References

File Name: Peer Review File

SUPPLEMENTARY FIGURES

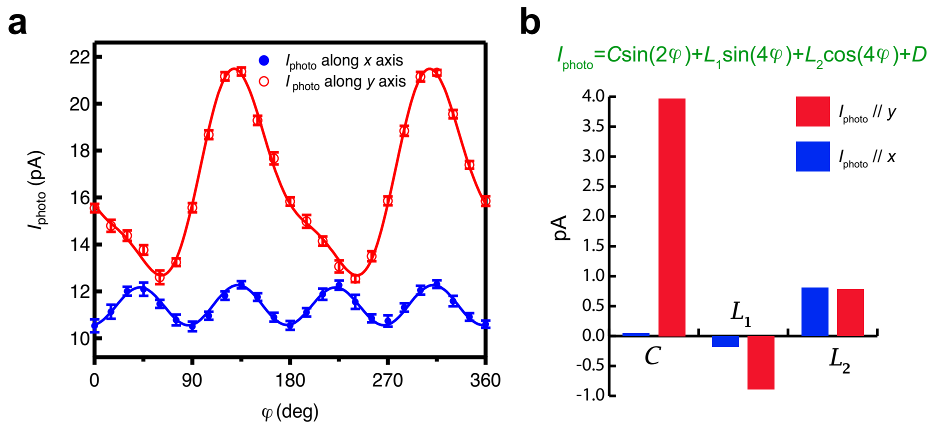

Supplementary Figure 1. The directionality of the helicity dependent photocurrent (HDPC) (a) The polarization dependent photocurrent in device E which was fabricated on the same thin film as device B with two identical conduction channels along x and y axis respectively. The laser is shed in the $x-z$ plane. Red and blue scatters denote the photocurrent along each conduction channel and are both fitted to Eq. (1) of the main text, denoted by the solid lines. (b) We extracted C, L_1 and L_2 from the Eq. (1) along x axis (blue) and y axis (red). The comparison between the two directions shows that C, the amplitude of the HDPC, diminishes along x axis. This demonstratse that the direction of the HDPC is perpendicular to the incidence plane. Besides, L_1 is significantly larger along y axis and L_2 is almost the same for the two directions, implying different origins between L_1 and L_2, which is consistent with the previous study.[1]

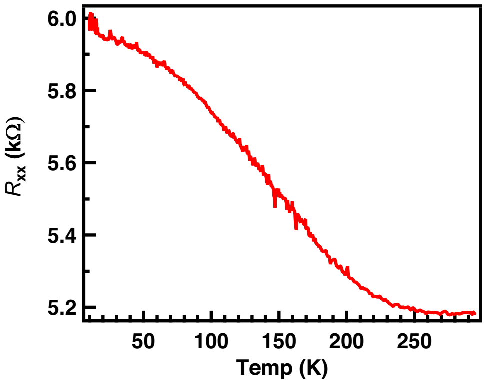

Supplementary Figure 2. Temperature dependence of the longitudinal resistance R_{xx} in device B. R_{xx} increases monotonically as we cool down the temperature from room temperature to 15K. The insulating behavior of R_{xx} provides another evidence that the chemical potential is indeed in the bulk band gap.

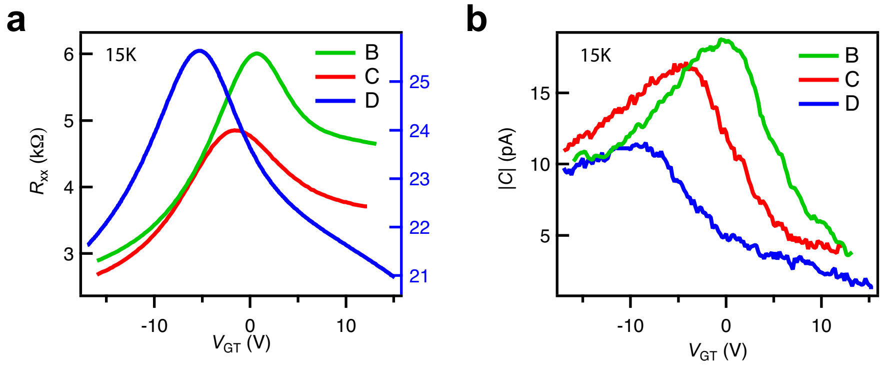

Supplementary Figure 3. Photocurrent in three $(Bi_{1-x}Sb_x)_2Te_3$ thin films with different Sb dopings. From the device B to D, we dope less Sb into Bi_2Te_3. As a consequence, the device D is n type while the chemical potential of device B is near the Dirac point. (a) The gate voltage dependence of the longitudinal resistance R_{xx}. The magnitude of R_{xx} in device D (blue curve), taken by a two probe measurement, is much larger than R_{xx} in devices B and C, taken by four probe measurements. Thus, we plot the R_{xx} of device D under a different scale on the right axis of the figure. The different peak positions of R_{xx} in devices B, C and D correspond to the Dirac points of B, C and D, respectively. (b) The gate voltage dependence of the absolute value of the HDPC amplitude– $|C|$. The peak position of the HDPC is in good agreement with the peak position of the R_{xx} in each device. Therefore, the observation that the HDPC maximizes when the chemical potential crosses the Dirac point, is repeatable and intrinsic for $(Bi_{1-x}Sb_x)_2Te_3$ thin films and independent of initial carrier concentrations, sample qualities, as well as other material details.

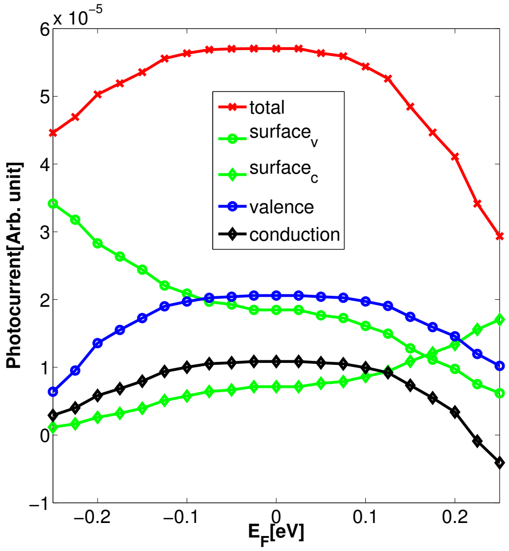

Supplementary Figure 4. Numerical calculation of photocurrent. Photocurrent contributed by excited carriers in different bands are separated. The red curve is the total photocurrent. The green curves are photocurrent induced by carriers in the surface states. They differ by the symbol and corresponds to photocurrent contributed by optical transitions from surface states to bulk conduction bands and from bulk valence bands to surface states, respectively. The blue curve is photocurrent induced by valence band carriers while the black curve is photocurrent induced by conduction band carriers. The magnitude of the photocurrent from valence bands is larger than that from conduction bands due to the large density of states for valence bands.

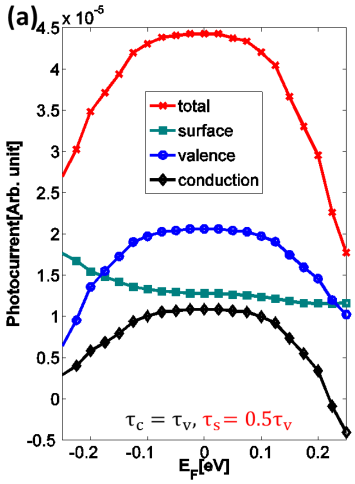

Supplementary Figure 5. Numerical calculation of photocurrent using different relaxation times for surface and bulk bands (a) Photocurrent for the case with $\tau_s = 0.5\tau_v$. τ_s, τ_c and τ_v denote the relaxation time of the surface states, conduction band and valence band respectively. (b) Photocurrent for the case with $\tau_s = \tau_v$. (c) Photocurrent for the case with $\tau_s = \tau_v$. Different relaxation times only change the results quantitatively while all the essential qualitative features, such as the peak at the Dirac point and the asymmetry between the electron and hole doping regimes, remain the same.

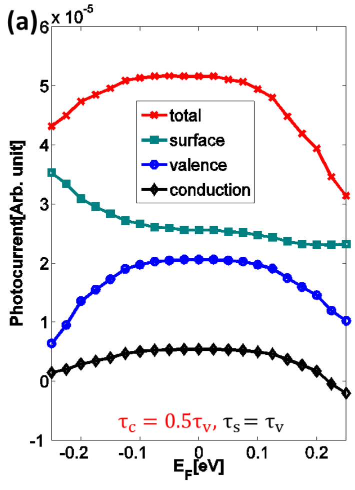

Supplementary Figure 6. Numerical calculation of photocurrent using different relaxation times for conduction and bulk bands (a) Photocurrent for the case with $\tau_c = 0.5\tau_v$. τ_s, τ_c and τ_v denote the relaxation time of the surface states, conduction band and valence band respectively. (b) Photocurrent for the case with $\tau_c = \tau_v$. (c) Photocurrent for the case with $\tau_c = \tau_v$. Different relaxation times only change the results quantitatively while all the essential qualitative features, such as the peak at the Dirac point and the asymmetry between the electron and hole doping regimes, remain the same.

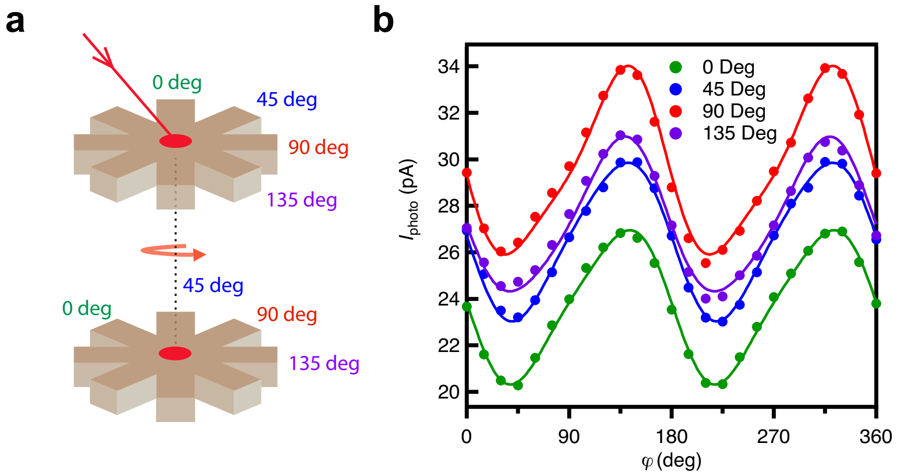

Supplementary Figure 7. Azimuthal angle dependence of the helicity dependent photocurrent (HDPC) (a) The schematic of the device F (fabricated on the same thin film as device A), which consists of four conduction channels along different crystal directions. They are noted by the relative angle of the channel direction. The laser is shed in the x-z plane and the photocurrent along each channel is measured after reorienting the channel along y axis. (b) The polarization dependent photocurrent along each channel. They are fitted to Eq. (1) and the extracted HDPC varies less than 10%. This demonstrates that the HDPC does not vary with the crystal direction along which the photocurrent is measured, consistent with the circular photo-galvanic effect on symmetry C_{3v}. We can also rule out the contribution from the surface shift current since the HDPC has a higher order of symmetry than the three-fold rotational symmetry. [2, 3]

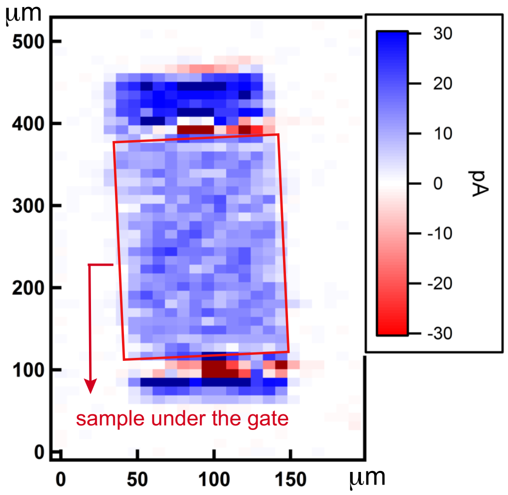

Supplementary Figure 8. Scanning photocurrent on device B with a 5 um wide He-Ne laser spot. The amplitude of the helicity dependent photocurrent -C- resampled at a step size of 10 um is shown. The blue color corresponds to a positive C while the red color denotes a negative C. Within the red box, the sample is covered by the top gate. At the top gate boundaries, we observe a large negative photocurrent due to the discontinuity of the gate material, manifested by the red stripes. Though we see some variation in C within the red box[4], the magnitude of C does not vary in a consistent way that suggests laser heating induced photocurrent.

SUPPLEMENTARY NOTE 1– SYMMETRY ARGUMENTS OF CPGE AND CPDE

CPGE and CPDE are both quadratic terms of electric field of light. Assume a monochromatic light shed in the $x-z$ plane has a wave vector $\mathbf{q}$, frequency ω, a polarization unit vector $\hat{e}$ and amplitude A. The electric field from the the light wave can be expressed as, $Aexp(-i\omega t+i\mathbf{qr})\hat{e}+c.c.$; the intensity of light $J=|A|^2$. The circular photogalvanic effect(CPGE) induced photocurrent can be expressed as $\mathbf{j}_i$=i$\gamma_{is}(\hat{e}\times\hat{e}^*)_s J$. The surface of the Bi-chalcogenides has a C_{3v} symmetry, corresponding to a three-fold rotation symmetry around z axis and three mirror planes in the $x-y$ plane. Under a rotation of $\frac{2\pi}{3}$, $\hat{e}\times\hat{e}^*$ becomes $\mathbf{R}(\hat{e}\times\hat{e}^*)$. $\mathbf{R}$ is the rotation matrix and equals to $\begin{pmatrix}\cos(\frac{2\pi}{3}) & \sin(\frac{2\pi}{3}) & 0\\ -\sin(\frac{2\pi}{3}) & \cos(\frac{2\pi}{3}) & 0\\ 0 & 0 & 1\end{pmatrix}$. $\mathbf{j}$ becomes $\mathbf{Rj}$. Because of the rotational symmetry, the tensor γ should remain the same. Therefore, we obtain that $\mathbf{Rj}$= i$\gamma\mathbf{R}(\hat{e}\times\hat{e}^*)$J. After replacing $\mathbf{j}$ with i$\gamma(\hat{e}\times\hat{e}^*)$J, we deduce that $\mathbf{R}\gamma=\gamma\mathbf{R}$. To satisfy this relation, γ must have a form of $\begin{pmatrix}\gamma_{xx} & \gamma_{xy} & 0\\ -\gamma_{xy} & \gamma_{xx} & 0\\ 0 & 0 & \gamma_{zz}\end{pmatrix}$. Furthermore, the mirror symmetry will impose an additional constraint on the form of γ. There are three mirror planes, and the angle between the mirror plane and the x axis is defined as ϕ. Under the mirror reflection, $\mathbf{j}$ becomes $\mathbf{Mj}$; however, as a pseudo-vector, $\hat{e}\times\hat{e}^*$ becomes -$\mathbf{M}(\hat{e}\times\hat{e}^*)$. $\mathbf{M}$ equals to $\begin{pmatrix}\cos(2\phi) & \sin(2\phi) & 0\\ \sin(2\phi) & -\cos(2\phi) & 0\\ 0 & 0 & 1\end{pmatrix}$. Therefore, $\mathbf{M}\gamma=-\gamma\mathbf{M}$. Combining the two constraints from mirror symmetry and rotational symmetry, we conclude that γ must have a form of $\begin{pmatrix}0 & \gamma_{xy} & 0\\ -\gamma_{xy} & 0 & 0\\ 0 & 0 & 0\end{pmatrix}$. Thus, $\mathbf{j}$=iJ $\begin{pmatrix}\gamma_{xy}(\hat{e}\times\hat{e}^*)_y\\ -\gamma_{xy}(\hat{e}\times\hat{e}^*)_x\\ 0\end{pmatrix}$.

The circular photon drag effect(CPDE) induced photocurrent can be expressed as $\mathbf{j}_i$=i$\tilde{T}_{ijl}\hat{q}_j(\hat{e}\times\hat{e}^*)_l J$. We define vector $\hat{h}\equiv\hat{e}\times\hat{e}^*$. For a transverse electromagnetic wave, the vector $\hat{h}$ should along the same direction as the photon's momentum $\hat{q}$. Therefore, $q_i h_j=q_j h_i$. We can define $\tilde{T}_{i1}\equiv\tilde{T}_{ixx}$, $\tilde{T}_{i2}\equiv\tilde{T}_{ixy}+\tilde{T}_{iyx}$, $\tilde{T}_{i3}\equiv\tilde{T}_{ixz}+\tilde{T}_{izx}$, $\tilde{T}_{i4}\equiv\tilde{T}_{iyy}$, $\tilde{T}_{i5}\equiv\tilde{T}_{iyz}+\tilde{T}_{izy}$ and $\tilde{T}_{i6}\equiv\tilde{T}_{izz}$. Under the rotation of $\frac{2\pi}{3}$, $j'=\mathbf{R}j=iJ\begin{pmatrix}R_{xx}\tilde{T}_{xij}+R_{xy}\tilde{T}_{yij}\\ R_{yx}\tilde{T}_{xij}+R_{yy}\tilde{T}_{yij}\\ \tilde{T}_{zij}\end{pmatrix}q_i h_j$. On the other hand, $j'=iJ\begin{pmatrix}\tilde{T}_{xij}R_{ik}q_k R_{js}h_s\\ \tilde{T}_{yij}R_{ik}q_k R_{js}h_s\\ \tilde{T}_{zij}R_{ik}q_k R_{js}h_s\end{pmatrix}$. For j'_z, we get $\tilde{T}_{zij}q_i h_j=\tilde{T}_{zij}R_{ik}q_k R_{js}h_s$. Each term–$q_i h_j$–needs to be equalized. Therefore, these conditions that $\tilde{T}_{z3}=\tilde{T}_{z5}=\tilde{T}_{z2}=0$ and $\tilde{T}_{z4}=\tilde{T}_{z1}$ need to be satisfied. Combined with the restrictions we get from j'_x and j'_y, we conclude that $\tilde{T}_{x1}$, $\tilde{T}_{x2}$, $\tilde{T}_{x3}$, $\tilde{T}_{x5}$, $\tilde{T}_{z1}$ and $\tilde{T}_{z6}$ are independent variables while $\tilde{T}_{x6}=\tilde{T}_{z2}=\tilde{T}_{z3}=\tilde{T}_{z5}=0$, $\tilde{T}_{x4}=-\tilde{T}_{x1}$, $\tilde{T}_{y1}=\frac{\sqrt{3}}{2}\tilde{T}_{x1}$, $\tilde{T}_{y2}=-2\tilde{T}_{x1}$, $\tilde{T}_{y3}=-\tilde{T}_{x5}$, $\tilde{T}_{y4}=-\frac{1}{2}\tilde{T}_{x2}$, $\tilde{T}_{y5}=\tilde{T}_{x3}$ and $\tilde{T}_{z4}=\tilde{T}_{z1}$. Besides, $\tilde{T}$ needs to satisfy mirror symmetry. Thus, $\begin{pmatrix}M_{xx}\tilde{T}_{xij}+M_{xy}\tilde{T}_{yij}\\ M_{yx}\tilde{T}_{xij}+M_{yy}\tilde{T}_{yij}\\ \tilde{T}_{zij}\end{pmatrix}q_i h_j=\begin{pmatrix}\tilde{T}_{xij}M_{ik}q_k(-M_{js}h_s)\\ \tilde{T}_{yij}M_{ik}q_k(-M_{js}h_s)\\ \tilde{T}_{zij}M_{ik}q_k(-M_{js}h_s)\end{pmatrix}$. Additional constraints on $\tilde{T}$ can be derived by matching each $q_i h_j$ term. Eventually we find that only $\tilde{T}_{y3}$=-$\tilde{T}_{x5}=\kappa$ are nonzero. Therefore, $\mathbf{j}$=i$J\begin{pmatrix}-\kappa q_z(\hat{e}\times\hat{e}^*)_y\\ \kappa q_z(\hat{e}\times\hat{e}^*)_x\\ 0\end{pmatrix}$.

For a monochromatic light shed in the $x-z$ plane, $(\hat{e}\times\hat{e}^*)_y=0$. Therefore, the photocurrent only flow along y direction for both CPGE and CPDE. Moreover, for an incidence angle of θ, $i(\hat{e}\times\hat{e}^*)_x=P\sin\theta$ and $q_z=-q\cos\theta$, where P stands for the helicity of the light. Therefore, $j_{\mathrm{CPGE}}=-\gamma_{xy}P\sin\theta J$ and $j_{\mathrm{CPDE}}=-\kappa Pq\sin\theta\cos\theta J$. Taken into account that $P=\sin(2\varphi)$, where φ denotes the angle between the fast axis of the quarter wave plate and the initial linear polarization of the light, we conclude that $j_{\mathrm{CPGE}}\propto\sin(2\varphi)\sin(\theta)$ and $j_{\mathrm{CPDE}}\propto\sin(2\varphi)\sin(2\theta)$.

SUPPLEMENTARY NOTE 2 – THEORETICAL ANALYSIS OF PHOTOCURRENT

Equation of photocurrent

We start the derivation of photocurrent equation based on the assumption that the momentum of light is small and can be ignored. Thus, only vertical optical transitions is considered. The photocurrent with a fixed photon energy

$\hbar\omega$ is expressed as

$$\mathbf{J} = -e\sum_{\eta}\sum_{\mathbf{k}}\mathbf{v}_{\mathbf{k},\eta}(f_{\mathbf{k},\eta} - f^0_{\mathbf{k},\eta}) \tag{1}$$

, where $\mathbf{k} = (k_x, k_y)$, e is the electron charge, η represents band index, $\mathbf{v}$ is the velocity of electrons for band η and $f_{\mathbf{k},\eta}(f^0_{\mathbf{k},\eta})$ is the equilibrium Fermi distribution function for band η with momentum $\mathbf{k}$ with(without) light excitation. We obtain the distribution function through the Boltzmann equation $\frac{df_{\mathbf{k},\eta}}{dt} = \sum_{\eta'} T_{\phi_{\mathbf{k},\eta'}\to\phi_{\mathbf{k},\eta}}(f^0_{\mathbf{k},\eta'} - f^0_{\mathbf{k},\eta}) + I_{\text{relax}}[f_{\mathbf{k},\eta}]$, where $T_{\phi_{\mathbf{k},\eta'}\to\phi_{\mathbf{k},\eta}} = \frac{2\pi}{\hbar}|\langle\phi_{\mathbf{k},\eta'}|H_{\text{int}}|\phi_{\mathbf{k},\eta}\rangle|^2\delta(E_{\mathbf{k},\eta} - E_{\mathbf{k},\eta'} - \hbar\omega)$ is the transition rate derived from Fermi's golden rule and $I_{\text{relax}}[f_{\mathbf{k},\eta}]$ describes the relaxation of excited carriers from states $\phi_{\mathbf{k},\eta}$. Under the assumption that the momentum relaxation time is much faster than the energy relaxation, we have $I_{\text{relax}}[f_{\mathbf{k},\eta}] = -\frac{f_{\mathbf{k},\eta} - f^0_{\mathbf{k},\eta}}{\tau_\eta}$, where τ_η is the momentum relaxation time of band η. When the system reaches its equilibrium state, $\frac{df_{\mathbf{k},\eta}}{dt} = 0$, we find that $f_{\mathbf{k},\eta} - f^0_{\mathbf{k},\eta} = \sum_{\eta'}\tau_\eta T_{\phi_{\mathbf{k},\eta'}\to\phi_{\mathbf{k},\eta}}(f^0_{\mathbf{k},\eta'} - f^0_{\mathbf{k},\eta})$. Thus, the photocurrent can be written as

$$\mathbf{J} = -\frac{2\pi e}{\hbar}\sum_{\mathbf{k},\langle\eta,\eta'\rangle}(\tau_\eta\mathbf{v}_{\mathbf{k},\eta} - \tau_{\eta'}\mathbf{v}_{\mathbf{k},\eta'})|\langle\phi_{\mathbf{k},\eta'}|H_{\text{int}}|\phi_{\mathbf{k},\eta}\rangle|^2(f^0_{\mathbf{k},\eta'} - f^0_{\mathbf{k},\eta})\delta(E_{\mathbf{k},\eta} - E_{\mathbf{k},\eta'} - \hbar\omega) \tag{2}$$

, where the summation $\langle\eta',\eta\rangle$ indicates pairs of the initial and final states. The interaction Hamiltonian H_{int} is derived as $-\frac{e}{\hbar}\frac{\partial H_0}{\partial\mathbf{k}}\cdot\mathbf{A}$ based on the minimal coupling $\mathbf{k}\to\Pi = \mathbf{k} - \frac{e}{\hbar}\mathbf{A}$ with $\mathbf{A}$ as the vector potential. Based on the $\mathbf{k}\cdot\mathbf{p}$ theory, one can obtain the Hamiltonian as $H_{\mathbf{k}\cdot\mathbf{p}} = (\epsilon_n + \frac{\hbar^2}{2m}\mathbf{k}^2)\delta_{nn'} + \frac{\hbar}{m}\mathbf{k}\cdot\mathbf{P}_{nn'}$, on the basis $|n\rangle$ with $n = 1,2,3...$ and $\mathbf{P}_{nn'} = \langle n|\hat{p}|n'\rangle$ describes the coupling between states $|n\rangle$ and $|n'\rangle$ with $\hat{p}$ as the momentum operator. Further, by using the second order perturbation to project irrelevant states $|l\rangle$ to the states we are considering, we can obtain an effective Hamiltonian $(H_{0,eff})_{nn'} = (\epsilon_n + \frac{\hbar^2}{2m}\mathbf{k}^2)\delta_{nn'} + \frac{\hbar}{m}\mathbf{k}\cdot\mathbf{P}_{nn'} + \frac{\hbar^2}{2m^2}\sum_{l\neq n,n'}(\mathbf{k}\cdot\mathbf{P}_{nl})(\mathbf{k}\cdot\mathbf{P}_{ln'})(\frac{1}{\epsilon_n - \epsilon_l} + \frac{1}{\epsilon'_n - \epsilon_l})$. Thus, the interaction Hamiltonian reads $(H_{\text{int}})_{nn'} = -\frac{e}{m}\mathbf{A}\cdot\mathbf{P}_{nn'} - \frac{e\hbar}{m}(\mathbf{A}\cdot\mathbf{k})\delta_{nn'} - \frac{e\hbar}{2m^2}\sum_{l\neq n,n'}((\mathbf{A}\cdot\mathbf{P}_{nl})(\mathbf{k}\cdot\mathbf{P}_{ln'}) + (\mathbf{k}\cdot\mathbf{P}_{nl})(\mathbf{A}\cdot\mathbf{P}_{ln'}))(\frac{1}{\epsilon_n - \epsilon_l} + \frac{1}{\epsilon'_n - \epsilon_l})$. In the small k limit, we only keep the k-independent term and obtain the interaction Hamiltonian as follows,

$$\hat{H}_{\text{int}} = -\frac{e}{m}\mathbf{A}\cdot\hat{\mathbf{p}} \tag{3}$$

For topological insulators(TIs), the bulk has inversion symmetry and will not contribute to the net HDPC. Therefore, we consider optical transitions between the bulk bands and the topological surface states. We use $\phi_{\mathbf{k},\eta}(z)$ and $\phi_{\mathbf{k},\eta'}(z)$ to denote the initial and the final states for an optical transition process. Thus, we have $H_0(z)\phi_{\mathbf{k},\eta}(z) = E_{\mathbf{k},\eta}\phi_{\mathbf{k},\eta}(z)$ and $H_0(z)\phi_{\mathbf{k},\eta'}(z) = E_{\mathbf{k},\eta'}\phi_{\mathbf{k},\eta'}(z)$. We further expand eigenstates of $H_0(z)$ in terms of basis $|n\rangle$ as $\phi_{\mathbf{k},\eta(\eta')}(z) = \sum_n d_{\eta(\eta')}(\mathbf{k},z)c_{\eta(\eta'),n}(\mathbf{k})|n\rangle$. Thus, we have $|\mathcal{M}|^2_{\eta\eta'} \equiv \langle\phi_{\mathbf{k},\eta'}|H_{\text{int}}|\phi_{\mathbf{k},\eta}\rangle = -\frac{e}{m}\mathbf{A}\cdot\sum_{z,n,n'}d^*_{\eta'}(\mathbf{k},z)d_\eta(\mathbf{k},z)c^*_{\eta',n}(\mathbf{k})c_{\eta,n'}(\mathbf{k})\langle n|\hat{p}|n'\rangle$. By denoting $\mathcal{D}_{\eta'\eta} = \frac{e}{m}\sum_{z,n,n'}d^*_{\eta'}(\mathbf{k},z)d_\eta(\mathbf{k},z)c^*_{\eta',n}(\mathbf{k})c_{\eta,n'}(\mathbf{k})\mathbf{P}_{nn'}$ with $\mathbf{P}_{nn'} = \langle n|\hat{p}|n'\rangle$, the photocurrent can be rewritten as

$$\mathbf{J} = -\frac{2\pi e}{\hbar}\sum_{\mathbf{k},\langle\eta,\eta'\rangle}(\tau_\eta\mathbf{v}_{\mathbf{k},\eta} - \tau_{\eta'}\mathbf{v}_{\mathbf{k},\eta'})|\mathcal{M}|^2_{\eta\eta'}(f^0_{\mathbf{k},\eta'} - f^0_{\mathbf{k},\eta})\delta(E_{\mathbf{k},\eta} - E_{\mathbf{k},\eta'} - \hbar\omega) \tag{4}$$

where $\mathcal{M}_{\eta\eta'} = \mathcal{D}\cdot\mathbf{A}$ is the matrix element for optical transitions from state $\phi_{\eta'}$ to ϕ_η.

Model Hamiltonian for Bi-chalcogenides

We learn from Ref. [5] that the bulk band eigenstates of Bi-chalcogenides in the double group of D^5_{3d} can only be in the $\Gamma^\pm_4$, $\Gamma^\pm_5$ and $\Gamma^\pm_6$ irreducible representation(IrRep), where $\pm$ labels the parity of the eigenstates. $\Gamma^\pm_{4,5}$ form states with angular momentum $j_z = \pm\frac{3}{2}$, while $\Gamma^\pm_6$ form states with angular momentum $j_z = \pm\frac{1}{2}$. States with $j_z = \pm\frac{3}{2}$ in $\Gamma^\pm_4$, $\Gamma^\pm_5$ IrRep can be expanded in terms of orbitals and spins as $|\Lambda_\pm, \frac{3}{2}\rangle = |\Lambda_\pm, p_+, \uparrow\rangle$ and $|\Lambda_\pm, -\frac{3}{2}\rangle = |\Lambda_\pm, p_-, \uparrow\rangle$, where Λ represents different combinations of atomic orbitals (Bi, Se, Sb), $|p_+\rangle = -\frac{1}{\sqrt{2}}(|p_x\rangle + i|p_y\rangle)$ and $|p_-\rangle = \frac{1}{\sqrt{2}}(|p_x\rangle - i|p_y\rangle)$. Furthermore, we express states in $\Gamma^\pm_{4,5}$ IrRep as $|\Lambda^\pm, \Gamma^\pm_4\rangle = \frac{1}{\sqrt{2}}(|\Lambda_\pm, \frac{3}{2}\rangle + |\Lambda_\pm, -\frac{3}{2}\rangle)$ and $|\Lambda^\pm, \Gamma^\pm_5\rangle = \frac{1}{\sqrt{2}}(|\Lambda_\pm, \frac{3}{2}\rangle - |\Lambda_\pm, -\frac{3}{2}\rangle)$. For states in $\Gamma^\pm_6$ IrRep, we have $|\Lambda_\pm, \frac{1}{2}\rangle = u_\Lambda|\Lambda_\pm, p_z, \uparrow\rangle + v_\Lambda|\Lambda_\pm, p_+, \downarrow\rangle$ and $|\Lambda_\pm, -\frac{1}{2}\rangle = u^*_\Lambda|\Lambda_\pm, p_z, \downarrow\rangle + v^*_\Lambda|\Lambda_\pm, p_-, \uparrow\rangle$, where $|\Lambda_\pm, -\frac{1}{2}\rangle$ is obtained from $|\Lambda_\pm, \frac{1}{2}\rangle$ by applying time reversal symmetry operation $\mathcal{T}$. Here we have used $\mathcal{T}|\Lambda_\pm\rangle = |\Lambda_\pm\rangle$, $\mathcal{T}|\uparrow\rangle = |\downarrow\rangle$, $\mathcal{T}|\downarrow\rangle = |\uparrow\rangle$, $\mathcal{T}|p_z\rangle = |p_z\rangle$, $\mathcal{T}|p_+\rangle = -|p_-\rangle$ and $\mathcal{T}|p_-\rangle = -|p_+\rangle$.

The states near the Fermi level belong to $\Gamma_6^{\pm}$ IrRep, which are denoted as $|P_1^+, \uparrow\rangle$, $|P_1^+, \downarrow\rangle$, $|P_2^-, \uparrow\rangle$ and $|P_2^-, \downarrow\rangle$, respectively. These states contribute to the nontrivial topological property[5, 6]. The effective Hamiltonian constructed from both symmetry principles and $\mathbf{k}\cdot\mathbf{p}$ theory[5, 6] is written as

$$H_1 = (M_0 + M_1 k_z^2)\tau_z \otimes \sigma_0 + B_0 k_z \tau_y \otimes \sigma_0 + A_0 \tau_x \otimes (\sigma_x k_y - \sigma_y k_x) \tag{5}$$

on the basis $\Psi = (|P_1^+, \uparrow\rangle, |P_1^+, \downarrow\rangle, |P_2^-, \uparrow\rangle, |P_2^-, \downarrow\rangle)^T$, where $\sigma_{0,x,y,z}$ and $\tau_{0,x,y,z}$ are unit/Pauli matrices in spin and orbital spaces, respectively. To simplify our calculation, here we omit the k_x^2 and k_y^2 related terms without changing the physics we are considering. For bulk states within the photon energy range that stay far away from $|P_1^+, \sigma\rangle$ and $|P_2^-, \sigma\rangle$ states in energy, we assume that the Hamiltonian takes a simple parabolic form.

$$H_2 = (E_b + \frac{\hbar^2}{2m_b^*}k^2)\sigma_0 \tag{6}$$

with m_b^* denoting the effective mass of bulk bands.

After the presentation of model Hamiltonian, we start to discuss the interaction between the surface states and the bulk states. More explicitly, we need to demonstrate how to calculate $\mathbf{P}_{nn'} = \langle n|\hat{p}|n'\rangle$. Possible bulk states $|n\rangle$ include $|P_3^+, \sigma\rangle$ and $|P_4^-, \sigma\rangle$ states in the $\Gamma_6^{\pm}$ irreducible representation and $|P_5^+, \pm\frac{3}{2}\rangle$, $|P_6^-, \pm\frac{3}{2}\rangle$ in the $\Gamma_{4,5}^{\pm}$ IrRep, where $P_{3,4,5,6}$ are notations for different possible bulk bands.

With the help of group theory and symmetry principle, we can figure out the nonzero interaction terms and also reduce the number of independent parameters. Recall that momentum operator $\hat{p}_z$ belongs to Γ_1^- IrRep and $\hat{p}_{\pm}$ belongs to Γ_3^- IrRep, where $\hat{p}_{\pm} \equiv \frac{1}{2}(\hat{p}_x \pm \hat{p}_y)$. Furthermore, $|P_{1,3}^-, \sigma\rangle$ belongs to Γ_6^- IrRep, $|P_{2,4}^+, \sigma\rangle$ belongs to Γ_6^+ IrRep, $|P_5^-, \pm\frac{3}{2}\rangle$ belong to $\Gamma_{4,5}^+$ IrRep and $|P_6^+, \pm\frac{3}{2}\rangle$ belong to $\Gamma_{4,5}^-$ IrRep. Moreover, the direct product of different representations is also useful, which is listed below.

$$\Gamma_6^s \times \Gamma_6^s = \Gamma_1^+ + \Gamma_2^+ + \Gamma_3^+ \tag{7}$$

$$\Gamma_6^s \times \Gamma_6^{-s} = \Gamma_1^- + \Gamma_2^- + \Gamma_3^- \tag{8}$$

$$\Gamma_6^+ \times \Gamma_{4,5}^{\pm} = \Gamma_3^{\pm} \tag{9}$$

$$\Gamma_6^- \times \Gamma_{4,5}^{\pm} = \Gamma_3^{\mp} \tag{10}$$

where $s = \pm$ denotes parity. Another useful tool we are using is the symmetry analysis. We denote the mirror symmetry operator as $\mathcal{M}_x = \mathcal{I} \otimes \mathcal{C}_{2,x}$ with yz plane as its mirror plane and time reversal symmetry operators $\mathcal{T}$, where $\mathcal{I}$ is the inversion operator and $\mathcal{C}_{2,x}$ is the two-fold rotational symmetry operator. Under the mirror and time reversal symmetry operations, the momentum operators transform as

$$\mathcal{M}_x \hat{p}_z \mathcal{M}_x^{-1} = \hat{p}_z \tag{11}$$

$$\mathcal{M}_x \hat{p}_+ \mathcal{M}_x^{-1} = -\hat{p}_x + i\hat{p}_y = -\hat{p}_- \tag{12}$$

$$\mathcal{M}_x \hat{p}_- \mathcal{M}_x^{-1} = -\hat{p}_x - i\hat{p}_y = -\hat{p}_+ \tag{13}$$

$$\mathcal{T} \hat{p}_z \mathcal{T}^{-1} = -\hat{p}_z \tag{14}$$

$$\mathcal{T} \hat{p}_+ \mathcal{T}^{-1} = -\hat{p}_x + (-i)(-\hat{p}_y) = -\hat{p}_- \tag{15}$$

$$\mathcal{T} \hat{p}_- \mathcal{T}^{-1} = -\hat{p}_x - (-i)(-\hat{p}_y) = -\hat{p}_+ \tag{16}$$

For bulk band states $|P_i^{\pm}, \sigma\rangle$ under the mirror symmetry transformation,

$$\mathcal{M}_x |P_{1,2,3,4}^{\pm}, \uparrow\rangle = i|P_{1,2,3,4}^{\pm}, \downarrow\rangle \tag{17}$$

$$\mathcal{M}_x |P_{1,2,3,4}^{\pm}, \downarrow\rangle = i|P_{1,2,3,4}^{\pm}, \uparrow\rangle \tag{18}$$

$$\mathcal{M}_x |P_{5,6}^{\pm}, \frac{3}{2}\rangle = i|P_{5,6}^{\pm}, -\frac{3}{2}\rangle \tag{19}$$

$$\mathcal{M}_x |P_{5,6}^{\pm}, -\frac{3}{2}\rangle = i|P_{5,6}^{\pm}, \frac{3}{2}\rangle \tag{20}$$

where $\mathcal{M}_x|P_{1,2,3,4,5,6}^{\pm}\rangle = |P_{1,2,3,4,5,6}^{\pm}\rangle$, $\mathcal{M}_x|\uparrow\rangle = i|\downarrow\rangle$, $\mathcal{M}_x|\downarrow\rangle = i|\uparrow\rangle$, $\mathcal{M}_x|p_z\rangle = |p_z\rangle$, $\mathcal{M}_x|p_+\rangle = |p_-\rangle$ and $\mathcal{M}_x|p_-\rangle = |p_+\rangle$ are used. Note that $P_{1,2,3,4,5,6}^{\pm}$ is just shorthand for $P_1^-, P_2^+, P_3^+, P_4^-, P_5^-, P_6^+$.

For bulk band states $|P_i^{\pm},\sigma\rangle$ under time reversal symmetry transformation, we have

$$\mathcal{T}|P_{1,2,3,4}^{\pm},\uparrow\rangle = |P_{1,2,3,4}^{\pm},\downarrow\rangle \tag{21}$$

$$\mathcal{T}|P_{1,2,3,4}^{\pm},\downarrow\rangle = -|P_{1,2,3,4}^{\pm},\uparrow\rangle \tag{22}$$

$$\mathcal{T}|P_{5,6}^{\pm},\frac{3}{2}\rangle = -|P_{5,6}^{\pm},-\frac{3}{2}\rangle \tag{23}$$

$$\mathcal{T}|P_{5,6}^{\pm},-\frac{3}{2}\rangle = |P_{5,6}^{\pm},+\frac{3}{2}\rangle \tag{24}$$

where $\mathcal{T}|P_{1,2,3,4,5,6}^{\pm}\rangle = |P_{1,2,3,4,5,6}^{\pm}\rangle$, $\mathcal{T}|\uparrow\rangle = |\downarrow\rangle$, $\mathcal{T}|\downarrow\rangle = -|\uparrow\rangle$, $\mathcal{T}|p_z\rangle = |p_z\rangle$, $\mathcal{T}|p_+\rangle = -|p_-\rangle$ and $\mathcal{T}|p_-\rangle = -|p_+\rangle$ are used. Now we are ready to calculate $\mathbf{P}_{nn'} = \langle n|\hat{p}|n'\rangle$.

$|P_3^+,\sigma\rangle$ bulk states in Γ_6^+ IrRep– Recall that $|P_3^+,\sigma\rangle$ belongs to Γ_6^+ IrRep, $\hat{p_z}$ belongs to Γ_1^- IrRep and $\hat{p}_\pm$ belongs to Γ_3^- IrRep. According to $\Gamma_6^s \times \Gamma_6^+ = \Gamma_1^s + \Gamma_2^s + \Gamma_3^s$, we know that only $|P_2^-,\sigma\rangle$ from the surface states make a contribution to $\mathbf{P}_{nn'}$. Moreover, there are only two independent terms in $\langle P_2^-,\sigma|\hat{\mathbf{p}}|P_3^+,\sigma'\rangle$. They are $\langle P_2^-,\uparrow|\hat{p}_z|P_3^+,\uparrow\rangle$ and $\langle P_2^-,\uparrow|\hat{p}_+|P_3^+,\downarrow\rangle$. The others are either forbidden by the C_3 symmetry or related through these two terms.

$$\langle P_2^-,\uparrow|\hat{p}_z|P_3^+,\uparrow\rangle = Q_3 \tag{25}$$

$$\langle P_2^-,\uparrow|\hat{p}_+|P_3^+,\downarrow\rangle = P_3 \tag{26}$$

, where $Q_3 = u_{P_2}^* u_{P_3}\langle P_2^-,p_z,\uparrow|(-i\hbar\partial_z)|P_3^+,p_z,\uparrow\rangle + v_{P_2}^* v_{P_3}\langle P_2^-,p_+,\downarrow|(-i\hbar\partial_z)|P_3^+,p_+,\downarrow\rangle$ and $P_3 = u_{P_2}^* v_{P_3}^*\langle P_2^-,p_z,\uparrow|(-i\hbar\partial_x + \hbar\partial_y)|P_3^+,p_-,\uparrow\rangle + v_{P_2}^* u_{P_3}^*\langle P_2^-,p_+,\downarrow|(-i\hbar\partial_x + \hbar\partial_y)|P_3^+,p_z,\downarrow\rangle$, which are material-dependent parameters.

The other two terms: $\langle P_2^-,\downarrow|\hat{p}_z|P_3^+,\downarrow\rangle, \langle P_2^-,\downarrow|\hat{p}_-|P_3^+,\uparrow\rangle$ can be obtained by either time reversal symmetry or mirror symmetry. Under mirror symmetry, $\langle P_2^-,\downarrow|\hat{p}_z|P_3^+,\downarrow\rangle = \langle P_2^-,\downarrow|\mathcal{M}_x^{-1}\mathcal{M}_x\hat{p}_z\mathcal{M}_x^{-1}\mathcal{M}_x|P_3^+,\downarrow\rangle = \langle \mathcal{M}_x P_2^-,\downarrow|\mathcal{M}_x\hat{p}_z\mathcal{M}_x^{-1}|\mathcal{M}_x P_3^+,\downarrow\rangle = \langle P_2^-,\uparrow|(-i)\hat{p}_z i|P_3^+,\uparrow\rangle = Q_3$. On the other hand, under time reversal symmetry, $\langle P_2^-,\downarrow|\hat{p}_z|P_3^+,\downarrow\rangle = \langle \mathcal{T}P_2^-,\uparrow|\hat{p}_z|\mathcal{T}P_3^+,\uparrow\rangle = -\langle \mathcal{T}P_2^-,\uparrow|\mathcal{T}(\hat{p}_z|P_3^+,\uparrow\rangle) = -\langle \hat{p}_z P_3^+,\uparrow|P_2^-,\uparrow\rangle = -\langle P_3^+,\uparrow|\hat{p}_z^\dagger|P_2^-,\uparrow\rangle = -Q_3^*$, where the property of anti-unitary of $\mathcal{T}$, $\langle \mathcal{T}\alpha|\mathcal{T}\beta\rangle = \langle \beta|\alpha\rangle$, is used. Thus, $Q_3 = -Q_3^*$, which is purely imaginary. Similarly, one can obtain$\langle P_2^-,\downarrow|\hat{p}_-|P_3^+,\uparrow\rangle = -P_3$ and $\langle P_2^-,\downarrow|\hat{p}_-|P_3^+,\uparrow\rangle = P_3^*$. Thus, $P_3 = -P_3^*$, which is also purely imaginary. In summary, we have

$$\langle P_2^-,\uparrow|\hat{p}_z|P_3^+,\uparrow\rangle = \langle P_2^-,\downarrow|\hat{p}_z|P_3^+,\downarrow\rangle = Q_3 = i\frac{m}{\hbar}B_3 \tag{27}$$

$$\langle P_2^-,\uparrow|\hat{p}_+|P_3^+,\downarrow\rangle = -\langle P_2^-,\downarrow|\hat{p}_-|P_3^+,\uparrow\rangle = P_3 = i\frac{m}{\hbar}A_3 \tag{28}$$

, where A_3 and B_3 are purely real. The term $\frac{m}{\hbar}$ is added in order to get A_3 and B_3 with unit of $eV\cdot$ Å. Moreover, $\langle P_3^+,\sigma|\hat{p}_z|P_2^-,\sigma\rangle$ can be obtained as

$$\langle P_3^+,\uparrow|\hat{p}_z|P_2^-,\uparrow\rangle = (\langle P_2^-,\uparrow|\hat{p}_z|P_3^+,\uparrow\rangle)^* = -i\frac{m}{\hbar}B_3 \tag{29}$$

$$\langle P_3^+,\downarrow|\hat{p}_-|P_2^-,\uparrow\rangle = (\langle P_2^-,\uparrow|\hat{p}_+|P_3^+,\downarrow\rangle)^* = -i\frac{m}{\hbar}A_3 \tag{30}$$

$$\langle P_3^+,\downarrow|\hat{p}_z|P_2^-,\downarrow\rangle = (\langle P_2^-,\downarrow|\hat{p}_z|P_3^+,\downarrow\rangle)^* = -i\frac{m}{\hbar}B_3 \tag{31}$$

$$\langle P_3^+,\uparrow|\hat{p}_+|P_2^-,\downarrow\rangle = (\langle P_2^-,\downarrow|\hat{p}_-|P_3^+,\uparrow\rangle)^* = i\frac{m}{\hbar}A_3 \tag{32}$$

Thus, the zeroth order of the light-matter interaction Hamiltonian $\hat{H}_{\text{int}}(P_3^+) = -\frac{e}{m}\mathbf{A}\cdot\hat{\mathbf{p}}$ in the small k limit on the basis $(|P_2^-,\uparrow\rangle, |P_2^-,\downarrow\rangle, |P_3^+,\uparrow\rangle, |P_3^+,\downarrow\rangle)^T$ is expressed as

$$H_{\text{int}}(P_3^+) = \frac{e}{\hbar}(B_3 A_z \tau_y \otimes \sigma_0 + A_3 A_x \tau_x \otimes \sigma_y - A_3 A_y \tau_x \otimes \sigma_x) \tag{33}$$

, where $A_{x,y,z}$ are x, y and z component of vector potential.

$|P_4^-,\sigma\rangle$ bulk states in Γ_6^- IrRep – The derivation for the coupling between $|P_4^-,\sigma\rangle$ states and surface states is the same as that for states $|P_3^+,\sigma\rangle$, except that the nonzero coupling terms come from $\langle P_1^+,\sigma|\hat{\mathbf{p}}|P_4^-,\sigma\rangle$. Here, we define that

$$\langle P_1^+,\uparrow|\hat{p}_z|P_4^-,\uparrow\rangle = \langle P_1^+,\downarrow|\hat{p}_z|P_4^-,\downarrow\rangle = i\frac{m}{\hbar}B_4 \tag{34}$$

$$\langle P_1^+,\uparrow|\hat{p}_+|P_4^-,\downarrow\rangle = -\langle P_1^+,\downarrow|\hat{p}_-|P_4^-,\uparrow\rangle = i\frac{m}{\hbar}A_4 \tag{35}$$

$$\langle P_4^-,\uparrow|\hat{p}_z|P_1^+,\uparrow\rangle = \langle P_4^-,\downarrow|\hat{p}_z|P_1^+,\downarrow\rangle = -i\frac{m}{\hbar}B_4 \tag{36}$$

$$\langle P_4^-,\uparrow|\hat{p}_+|P_1^+,\downarrow\rangle = -\langle P_4^-,\downarrow|\hat{p}_-|P_1^+,\uparrow\rangle = i\frac{m}{\hbar}A_4 \tag{37}$$

, where $B_4 = \frac{\hbar}{m} u^*_{P_1} u_{P_4} \langle P_1^+, p_z, \uparrow |(-\hbar\partial_z)|P_4^-, p_z, \uparrow\rangle + \frac{\hbar}{m} v^*_{P_1} v_{P_4} \langle P_1^+, p_+, \downarrow |(-\hbar\partial_z)|P_4^-, p_+, \downarrow\rangle$ and $A_4 = \frac{\hbar}{m} u^*_{P_1} v^*_{P_4} \langle P_1^+, p_z, \uparrow |(-\hbar\partial_x - i\hbar\partial_y)|P_4^-, p_-, \uparrow\rangle + \frac{\hbar}{m} v^*_{P_1} u^*_{P_4} \langle P_1^+, p_+, \downarrow |(-\hbar\partial_x - i\hbar\partial_y)|P_4^-, p_z, \downarrow\rangle$, which are material-dependent parameters.

Thus, the zeroth order of the light-matter interaction Hamiltonian $\hat{H}_{\text{int}}(P_4^-) = -\frac{e}{m}\mathbf{A}\cdot\hat{\mathbf{p}}$ in the small k limit on the basis $(|P_1^+, \uparrow\rangle, |P_1^+, \downarrow\rangle, |P_4^-, \uparrow\rangle, |P_4^-, \downarrow\rangle)^T$ is expressed as

$$H_{\text{int}}(P_4^-) = \frac{e}{\hbar}(B_4 A_z \tau_y \otimes \sigma_0 + A_4 A_x \tau_x \otimes \sigma_y - A_4 A_y \tau_x \otimes \sigma_x) \tag{38}$$

<u>$|P_5^-, \sigma\rangle$ *bulk states in* $\Gamma_{4,5}^-$ *IrRep* –</u> Recall that $|P_5^-, \pm\frac{3}{2}\rangle$ belongs to $\Gamma_{4,5}^-$ IrRep and $\hat{p}_\pm$ belongs to Γ_3^- IrRep. According to $\Gamma_5^s \times \Gamma_{4,5}^- = \Gamma_3^s$, we know that only $|P_1^+, \sigma\rangle$ components in the surface states contribute to the matrix element. Moreover, there are only two independent terms in $\langle P_1^+, \sigma|\hat{\mathbf{p}}|P_5^-, \pm\frac{3}{2}\rangle$: $\langle P_1^+, \uparrow |\hat{p}_+|P_5^-, \frac{3}{2}\rangle$ and $\langle P_1^+, \uparrow |\hat{p}_-|P_5^-, -\frac{3}{2}\rangle$. The other terms are either forbidden by the C_3 rotational symmetry or related through these two terms. We assume

$$\langle P_1^+, \uparrow |\hat{p}_-|P_5^-, \frac{3}{2}\rangle = Q_5 \tag{39}$$

$$\langle P_1^+, \uparrow |\hat{p}_-|P_5^-, -\frac{3}{2}\rangle = P_5 \tag{40}$$

, where $Q_5 = u^*_{P_1} \langle P_1^+, p_z, \uparrow |(-i\hbar\partial_x - \hbar\partial_y)|P_5^-, p_+, \uparrow\rangle$ and $P_5 = v^*_{P_1} \langle P_1^+, p_+, \downarrow |(-i\hbar\partial_x - \hbar\partial_y)|P_5^-, p_-, \downarrow\rangle$, which are material-dependent parameters. The other two terms, $\langle P_1^+, \downarrow |\hat{p}_+|P_5^-, -\frac{3}{2}\rangle, \langle P_1^+, \downarrow |\hat{p}_+|P_5^-, \frac{3}{2}\rangle$ can be obtained by either time reversal symmetry or mirror symmetry. For instance, $\langle P_1^+, \downarrow |\hat{p}_+|P_5^-, -\frac{3}{2}\rangle = \langle P_1^+, \downarrow |\mathcal{M}_x^{-1}\mathcal{M}_x \hat{p}_+ \mathcal{M}_x^{-1}\mathcal{M}_x|P_5^-, -\frac{3}{2}\rangle = \langle \mathcal{M}_x P_1^+, \downarrow |\mathcal{M}_x \hat{p}_+ \mathcal{M}_x^{-1}|\mathcal{M}_x P_5^-, -\frac{3}{2}\rangle = \langle P_1^+, \uparrow |(-i)(-\hat{p}_-)i|P_5^-, \frac{3}{2}\rangle = -Q_5$. On the other hand, $\langle P_1^+, \downarrow |\hat{p}_+|P_5^-, -\frac{3}{2}\rangle = \langle \mathcal{T}P_1^+, \uparrow |\hat{p}_+|(-1)\mathcal{T}P_5^-, \frac{3}{2}\rangle = \langle \mathcal{T}P_1^+, \uparrow |\mathcal{T}(\hat{p}_-|P_5^-, \frac{3}{2}\rangle) = \langle \hat{p}_- P_5^-, \frac{3}{2}|P_1^+, \uparrow\rangle = \langle P_5^-, \frac{3}{2}|\hat{p}_-^\dagger|P_1^+, \uparrow\rangle = Q_5^*$. Thus, $Q_5 = -Q_5^*$, which is purely imaginary.

Similarly, one can obtain $\langle P_1^+, \downarrow |\hat{p}_+|P_5^-, \frac{3}{2}\rangle = -P_5$ and $\langle P_1^+, \downarrow |\hat{p}_+|P_5^-, \frac{3}{2}\rangle = -P_5^*$. Thus, $P_5 = P_5^*$, which is also purely real. In summary, we have

$$\langle P_1^+, \uparrow |\hat{p}_-|P_5^-, \frac{3}{2}\rangle = -\langle P_1^+, \downarrow |\hat{p}_+|P_5^-, -\frac{3}{2}\rangle = Q_5 = i\frac{m}{\hbar}B_5 \tag{41}$$

$$\langle P_1^+, \uparrow |\hat{p}_-|P_5^-, -\frac{3}{2}\rangle = -\langle P_1^+, \downarrow |\hat{p}_+|P_5^-, \frac{3}{2}\rangle = P_5 = \frac{m}{\hbar}A_5 \tag{42}$$

, where A_5 and B_5 are purely real. Similarly, we have

$$\langle P_5^-, \frac{3}{2}|\hat{p}_+|P_1^+, \uparrow\rangle = -\langle P_5^-, -\frac{3}{2}|\hat{p}_-|P_1^-, \downarrow\rangle = -i\frac{m}{\hbar}B_5 \tag{43}$$

$$\langle P_5^-, -\frac{3}{2}|\hat{p}_+|P_1^+, \uparrow\rangle = -\langle P_5^-, \frac{3}{2}|\hat{p}_-|P_1^+, \downarrow\rangle = \frac{m}{\hbar}A_5 \tag{44}$$

Thus, the zeroth order of the light-matter interaction Hamiltonian $\hat{H}_{\text{int}}(P_5^-) = -\frac{e}{m}\mathbf{A}\cdot\hat{\mathbf{p}}$ in the small k limit on the basis $(|P_1^+, \uparrow\rangle, |P_1^+, \downarrow\rangle, |P_5^-, \frac{3}{2}\rangle, |P_5^-, -\frac{3}{2}\rangle)^T$ is expressed as

$$H_{\text{int}}(P_5^-) = \frac{e}{\hbar}(B_5 A_x \tau_y \otimes \sigma_z + B_5 A_y \tau_x \otimes \sigma_0 + A_5 A_x \tau_y \otimes \sigma_y + A_5 A_y \tau_y \otimes \sigma_x) \tag{45}$$

<u>$|P_6^+, \sigma\rangle$ *bulk states in* $\Gamma_{4,5}^+$ *IrRep* –</u> The derivation of coupling is the same as the case for $|P_5^-, \sigma\rangle$ bulk states, except that the nonzero coupling terms come from $\langle P_2^-, \sigma|\hat{\mathbf{p}}|P_6^+, \sigma\rangle$. Similarly, we have

$$r\langle P_2^-, \uparrow |\hat{p}_-|P_6^+, \frac{3}{2}\rangle = -\langle P_2^-, \downarrow |\hat{p}_+|P_6^+, -\frac{3}{2}\rangle = i\frac{m}{\hbar}B_6 \tag{46}$$

$$\langle P_2^-, \uparrow |\hat{p}_-|P_6^+, -\frac{3}{2}\rangle = -\langle P_2^-, \downarrow |\hat{p}_+|P_6^+, \frac{3}{2}\rangle = \frac{m}{\hbar}A_6 \tag{47}$$

$$\langle P_6^+, \frac{3}{2}|\hat{p}_+|P_2^-, \uparrow\rangle = -\langle P_6^+, -\frac{3}{2}|\hat{p}_-|P_2^-, \downarrow\rangle = -i\frac{m}{\hbar}B_6 \tag{48}$$

$$\langle P_6^+, -\frac{3}{2}|\hat{p}_+|P_2^-, \uparrow\rangle = -\langle P_6^+, \frac{3}{2}|\hat{p}_-|P_2^-, \downarrow\rangle = \frac{m}{\hbar}A_6 \tag{49}$$

, where $i\frac{m}{\hbar}B_6 = u^*_{P_2} \langle P_2^-, p_z, \uparrow |(-i\hbar\partial_x - \hbar\partial_y)|P_6^+, p_+, \uparrow\rangle$ and $\frac{m}{\hbar}A_6 = v^*_{P_2} \langle P_2^-, p_+, \downarrow |(-i\hbar\partial_x - \hbar\partial_y)|P_6^+, p_-, \downarrow\rangle$, which are material-dependent parameters. A_5 and B_5 are purely real.

Thus, the zeroth order of the light-matter interaction Hamiltonian $\hat{H}_{\text{int}}(P_6^+) = -\frac{e}{m}\mathbf{A}\cdot\hat{\mathbf{p}}$ in the small k limit on the basis $(|P_2^-, \uparrow\rangle, |P_2^-, \downarrow\rangle, |P_6^+, \frac{3}{2}\rangle, |P_6^+, -\frac{3}{2}\rangle)^T$ is expressed as

$$H_{\text{int}}(P_6^+) = \frac{e}{\hbar}(B_6 A_x \tau_y \otimes \sigma_z + B_6 A_y \tau_x \otimes \sigma_0 + A_6 A_x \tau_y \otimes \sigma_y + A_6 A_y \tau_y \otimes \sigma_x) \tag{50}$$

SUPPLEMENTARY NOTE 3 – DERIVATION OF OPTICAL TRANSITION MATRIX AND PHOTOCURRENT FOR BI-CHALCOGENIDES

In the following section, we will derive the matrix element and photocurrent for each possible bulk band. We start the derivation with introducing the wavefunctions of surface states and bulk states for a thin film of Bi-chalcogenides.

At Γ point $k_x = k_y = 0$ in the momentum, one can solve the Hamiltonian for the topological insulators, as shown in Eq. 5 in $z > 0$ space by replacing $k_z \to -i\partial_z$. The two states can be expressed as $|\Psi_\sigma\rangle = \frac{f(z)}{\sqrt{2}}(|P_1^+, \sigma\rangle + |P_2^-, \sigma\rangle)$, where $f(z)$ is a layer-dependent parameter and σ denotes spin up or spin down. We can see that the states with opposite parities couple to each other because of the inversion symmetry breaking for the surface. By projecting the bulk Hamiltonian of Eq. 5 onto the subspace of these two states $|\Psi_{s,z}\rangle = (|\Psi_\uparrow\rangle, |\Psi_\downarrow\rangle)^T$ with $k_z \to -i\partial_z$, we arrive at the effective Hamiltonian of the surface states, which is written as

$$H_s = A_0(k_y\sigma_x - k_x\sigma_y) \tag{51}$$

where $A_0 > 0$. The velocity of surface states has an order of magnitude $v = A_0/\hbar \sim 10^5 m/s$[6]. Furthermore, one can solve for the two eigenstates of the topological surface states, expressed as $\phi_{s,\mathbf{k},\xi} = \frac{1}{\sqrt{2}}(i\xi e^{-i\theta_k}, 1)^T$ with eigenenergy $E_{s,k,\xi} = A_0\xi|k|$, where $\xi = \pm 1$ labels the upper(lower) Dirac cone with left-handed(right-handed) spin texture, and $\theta_k = tan^{-1}(\frac{k_y}{k_x})$. Concretely, we expand the eigenstates $\phi_{s,\mathbf{k},\xi}$ on the basis $\Psi_s(z) = (|P_1^+, \uparrow\rangle, |P_1^+, \downarrow\rangle, |P_2^-, \uparrow\rangle, |P_2^-, \downarrow\rangle)^T$, which is expressed as

$$\phi_{s,\mathbf{k},\xi} = \frac{f(z)}{2}(i\xi e^{-i\theta_k}, 1, i\xi e^{-i\theta_k}, 1)^T \tag{52}$$

For the bulk states, one can rearrange the eigenstates with in-plane left-handed and right-handed spin texture due to the degeneracy of bulk states. Explicitly, the rearranged bulk eigenstates are expressed as

$$\phi_{b,\mathbf{k},\eta} = \frac{1}{\sqrt{2}}(i\eta e^{-i\theta_k}, 1)^T \tag{53}$$

on the basis $\Psi_b(z) = g(z)(|\Lambda_i^\pm, \uparrow(\frac{3}{2})\rangle, |\Lambda_i^\pm, \downarrow(-\frac{3}{2})\rangle)^T$, where $\eta = \pm 1$ represents the in-plane left-handed and right-handed spin texture. Here we would like to mention that ξ and η always label surface states and bulk states, respectively.

P_3^+ bulk states

1. *Optical transitions from valence bands to surface bands*

Recall that the matrix element for optical transitions from valence bands to surface bands is $|\mathcal{M}|^2_{\xi\eta} = |\mathbf{A}\cdot\mathcal{D}_{\xi\eta}|^2$ where $\mathcal{D}_{\xi\eta} = \frac{e}{m}\sum_{z,n,n'} d^*_\xi(\mathbf{k},z)d_\eta(\mathbf{k},z)c^*_{\xi,n}(\mathbf{k})c_{\eta,n'}(\mathbf{k})\mathbf{P}_{nn'}$. We first calculate $\mathcal{D}_{\xi\eta}$. Here the parameters are taken as $d_\xi = \frac{1}{\sqrt{2}}f(z)$, $d_\eta = g(z)$, $c_{\xi,|P_2^-,\uparrow\rangle} = \frac{1}{\sqrt{2}}i\xi e^{-i\theta_k}$, $c_{\xi,|P_2^-,\downarrow\rangle} = \frac{1}{\sqrt{2}}$, $c_{\eta,|P_3^+,\uparrow\rangle} = \frac{1}{\sqrt{2}}i\eta e^{-i\theta_k}$ and $c_{\eta,|P_3^+,\downarrow\rangle} = \frac{1}{\sqrt{2}}$. Thus, we have $\mathcal{D}^z_{\xi\eta}(v\to s) = \frac{e}{m}\sum_z \frac{1}{\sqrt{2}}f^*(z)g(z)(c^*_{\xi,|P_2^-,\uparrow\rangle}c_{\eta,|P_3^+,\uparrow\rangle}i\frac{m}{\hbar}B_3 + c^*_{\xi,|P_2^-,\downarrow\rangle}c_{\eta,|P_3^+,\downarrow\rangle}i\frac{m}{\hbar}B_3) = \frac{ieF_3}{2\hbar}(\eta\xi B_3 + B_3)$, where $F_3 = \sum_z \frac{1}{\sqrt{2}}f^*(z)g(z)$, which is independent of the momentum by using small k approximation. Similarly, $\mathcal{D}^+_{\xi\eta}(v\to s) = \frac{e}{m}\sum_z \frac{1}{\sqrt{2}}f^*(z)g(z)(c^*_{\xi,|P_2^-,\uparrow\rangle}c_{\eta,|P_3^+,\downarrow\rangle}i\frac{m}{\hbar}A_3) = \frac{eF_3}{2\hbar}(\xi e^{i\theta_k}A_3)$ and $\mathcal{D}^-_{\xi\eta}(v\to s) = \frac{e}{m}\sum_z \frac{1}{\sqrt{2}}f^*(z)g(z)(c^*_{\xi,|P_2^-,\downarrow\rangle}c_{\eta,|P_3^+,\uparrow\rangle}(-i\frac{m}{\hbar}A_3)) = \frac{eF_3}{2\hbar}(\eta e^{-i\theta_k}A_3)$. Concretely,

$$\mathcal{D}_{\xi\eta}(v\to s) = \frac{ieF_3}{2\hbar}[(\eta\xi B_3 + B_3)\hat{e}_z + (-i\eta e^{-i\theta_k}A_3 - i\xi e^{i\theta_k}A_3)/\sqrt{2}\hat{e}_x + (\eta e^{-i\theta_k}A_3 - \xi e^{i\theta_k}A_3)/\sqrt{2}\hat{e}_y] \tag{54}$$

, where $\mathcal{D}^+_{\xi\eta}(v\to s) = \frac{1}{\sqrt{2}}(\mathcal{D}^x_{\xi\eta} + i\mathcal{D}^y_{\xi\eta})$ and $\mathcal{D}^-_{\xi\eta}(v\to s) = \frac{1}{\sqrt{2}}(\mathcal{D}^x_{\xi\eta} - i\mathcal{D}^y_{\xi\eta})$ are used.

We assume the incident light towards the surface of Bi-chalcogenides has polar angle θ and azimuthal angle γ. The wavevector $\mathbf{q}$ can be expressed as $\mathbf{q} = -q(\sin(\theta)\cos(\gamma), \sin(\theta)\sin(\gamma), \cos(\theta))$. Furthermore, we obtain the vector potential as $\mathbf{A}(t) = A_E e^{-i\omega t + i\mathbf{q}\cdot\mathbf{r}}(-i\sin(2\varphi)\sin(\gamma) + (1 - i\cos(2\varphi))\cos(\theta)\cos(\gamma), i\sin(2\varphi)\cos(\gamma) + (1 - i\cos(2\varphi))\cos(\theta)\sin(\gamma), -(1 - i\cos(2\varphi))\sin(\theta)) + c.c.$, where $A_E = \frac{E_0}{i\omega}$ with E_0 as the magnitude of the applied electric field and φ is the angle between the fast axis and the initial linear polarization of the light. The first term describes the photon absorption process while the second term describes photon emission process. For our experimental setup, $\gamma = \pi$ and $\varphi = \frac{\pi}{4}, \frac{3\pi}{4}$ for left/right circularly polarized light. Thus, for an incident light, $A_x = -(1 - i\cos(2\varphi))\cos(\theta)A_E$, $A_y = -i\sin(2\varphi)A_E$ and $A_z = -(1 - i\cos(2\varphi))\sin(\theta)A_E$.

Now, we are ready to calculate the matrix element $|\mathcal{M}|^2_{\xi\eta}(v \to s)$.

$$|\mathcal{M}|^2_{\xi\eta}(v \to s) = |-(1-i\cos(2\varphi))\cos(\theta)A_E\mathcal{D}^x_{\xi\eta} - i\sin(2\varphi)A_E\mathcal{D}^y_{\xi\eta} - (1-i\cos(2\varphi))\sin(\theta)A_E D^z_{\xi\eta}|^2 \quad (55)$$

$$= \frac{e^2F_3^2A_E^2}{4\hbar^2}[A_3^2\cos^2(\theta)(1+\xi\eta(1+\cos^2(2\varphi))\cos(2\theta_k)) + A_3^2\sin^2(2\varphi)(1-\xi\eta\cos(2\theta_k)) + B_3^2\sin^2(\theta)(1+\cos^2(2\varphi))$$
$$(1+\xi\eta)^2 + \sqrt{2}A_3B_3\sin(2\varphi)\sin(\theta)\eta(1+\eta\xi)^2\sin(\theta_k)] \quad (56)$$

Since the velocity $\mathbf{v}_{\mathbf{k},\eta}$ is odd in terms of $\mathbf{k}$ in the momentum space, only terms in $|\mathcal{M}|^2_{\xi\eta}$ that are asymmetric with respect to $\mathbf{k}$ contribute to nonzero photocurrent. Therefore, the asymmetric part of matrix element, denoted as $|\mathcal{M}|^2_{\xi\eta,a}$, is expressed as

$$|\mathcal{M}|^2_{\xi\eta,a}(v \to s) = \Delta_{3,M}\sin(2\varphi)\sin(\theta)\sin(\theta_k)\xi\delta_{\xi\eta} \quad (57)$$

where $\Delta_{3,M} = \frac{\sqrt{2}e^2F_3^2A_E^2}{\hbar^2}A_3B_3$ in unit of eV, $\xi = \pm 1$ indicates upper/lower Dirac cone with left-handed/right-handed spin texture and $\eta = \pm 1$ indicates bulk eigenstates with left-handed/right-handed spin texture.

We can obtain the following conclusions from the above expression for the asymmetric part of the matrix element. 1, $|\mathcal{M}|^2_{\xi\eta,a}$ depends on the helicity of the light; 2, The magnitude of $|\mathcal{M}|^2_{\xi\eta,a}$ is proportional to $\sin(\theta)$; 3, The nontrivial contribution to $|\mathcal{M}|^2_{\xi\eta,a}$ comes from optical transitions between surface states and bulk states with the same in-pane spin($\xi = \eta$); 4, $|\mathcal{M}|^2_{\xi\eta,a}$ depends on the Dirac cone index ξ. The asymmetric part of matrix element contributed from the upper Dirac cone is opposite from that for the lower Dirac cone at the same momentum $\mathbf{k}$. 6, $|\mathcal{M}|^2_{\xi\eta,a}$ depends on the sign of A_3B_3, which depend on the property of different bulk states; 7, The $|\mathcal{M}|^2_{\xi\eta,a}$ is zero along line $k_y = 0$.

Now let us start the derivation of photocurrent from the bulk states $\phi_{b,\mathbf{k},\eta}$ with

$$\mathbf{J}(v \to s) = -\frac{2\pi e}{\hbar}\sum_{\mathbf{k},\langle\xi,\eta\rangle}(\tau_\xi\mathbf{v}_{\mathbf{k},\xi} - \tau_\eta\mathbf{v}_{\mathbf{k},\eta})|\mathcal{M}|^2_{\xi\eta,a}(f^0_{\mathbf{k},\eta} - f^0_{\mathbf{k},\xi})\delta(E_{\mathbf{k},\xi} - E_{\mathbf{k},\eta} - \hbar\omega) = \mathbf{J}_s(v \to s) + \mathbf{j}_b(v \to s) \quad (58)$$

where $\mathbf{J}_s(v \to s) = -\frac{2\pi e\tau_s}{\hbar}\sum_{\mathbf{k},\langle\xi,\eta\rangle}\mathbf{v}_{s,\mathbf{k},\xi}|\mathcal{M}|^2_{\xi\eta,a}(f^0_{\mathbf{k},\eta} - f^0_{\mathbf{k},\xi})\delta(E_{\mathbf{k},\xi} - E_{\mathbf{k},\eta} - \hbar\omega)$ is the photocurrent contributed from the surface states and $\mathbf{J}_b(v \to s) = \frac{2\pi e\tau_v}{\hbar}\sum_{\mathbf{k},\langle\xi,\eta\rangle}\mathbf{v}_{b,\mathbf{k},\eta}|\mathcal{M}|^2_{\xi\eta,a}(f^0_{\mathbf{k},\eta} - f^0_{\mathbf{k},\xi})\delta(E_{\mathbf{k},\xi} - E_{\mathbf{k},\eta} - \hbar\omega)$ is the photocurrent contributed from the bulk states, where τ_s and τ_v represent relaxation time for surface and bulk carriers, respectively. Recall $E_{s,k,\xi} = A_0\xi|k|$ and $E_{b,k,\xi} = E_b - M_vk^2$ with $M_v \equiv \frac{\hbar^2}{2m_v^*}$. Therefore, $\mathbf{v}_{s,\mathbf{k},\xi} = \frac{\partial E_{s,k,\xi}}{\hbar\partial|k|}\frac{\partial|k|}{\partial k_x}\hat{e}_x + \frac{\partial E_{s,k,\xi}}{\hbar\partial|k|}\frac{\partial|k|}{\partial k_y}\hat{e}_y = \frac{A_0}{\hbar}\xi\cos(\theta_k)\hat{e}_x + \frac{A_0}{\hbar}\xi\sin(\theta_k)\hat{e}_y$ and $\mathbf{v}_{b,\mathbf{k},\eta} = -\frac{2}{\hbar}M_v|k|\cos(\theta_k)\hat{e}_x - \frac{2}{\hbar}M_v|k|\sin(\theta_k)\hat{e}_y$. We then calculate $\mathbf{J}_s$ and $\mathbf{J}_b$ by substituting the matrix element with the expression derived above. After steps of mathematical calculation, we arrive at

$$\mathbf{J}_s(E_F, v \to s) = -\frac{e^3F_3^2A_E^2A_3B_3\sin(2\varphi)\sin(\theta)\tau_sS_s^2}{16\sqrt{2}\pi^2\hbar^4A_0M_v}[A_0^2k_c^2 - sgn(E_F)E_F^2]\hat{e}_y \quad (59)$$

Where E_F is the Fermi energy, k_c is the cutoff momentum, $sgn(E_F)$ denotes the sign of E_F and S_s can be regarded as the size of sample. We also assume that the condition $E_{\mathbf{k},\xi} - E_{\mathbf{k},\eta} - \hbar\omega = 0$ can be always fulfilled and the temperature is $T = 0$K during the derivation process. Here we have also used the density of states for Dirac-cone surface states $g(E_\xi, \theta_k) = S_sg(k,\theta_k)dk/dE_\xi = S_s|\frac{kdk}{4\pi^2dE_\xi}| = |\frac{S_sE_\xi}{4\pi^2A_0^2}|$.

$$\mathbf{J}_b(E_F, v \to s) = -\frac{e^3F_3^2A_E^2A_3B_3\sin(2\varphi)\sin(\theta)\tau_vS_s^2}{4\sqrt{2}\pi^2\hbar^4A_0^2\sqrt{M_v}}(E_v + \hbar\omega - E_F)^{\frac{3}{2}}(\frac{4}{15}(E_v + \hbar\omega) + \frac{2}{5}E_F)\hat{e}_y \quad (60)$$

One can easily check that the maximum of function $f(x) = (a-x)^{\frac{3}{2}}(\frac{4}{15}a + \frac{2}{5}x)$ happens when $x = 0$. Thus, the magnitude of $\mathbf{J}_b(E_F, v \to s)$ takes its maximum when $E_F = 0$. It decays as the Fermi level moves away from the Dirac point.

We can obtain the following conclusions from the above expression for the photocurrent. 1, Photocurrent can only exist along y direction; 2, The sign of the photocurrents originates from $sgn(-A_3B_3\sin(2\varphi))$ for both $\mathbf{J}_s$ and $\mathbf{J}_b$; 3, The magnitude of photocurrent, which is proportional to $\sin(\theta)$, depends on the polar angle of incident light; 4, Only optical transition between surface states and bulk states with the same in-plane spin texture make a nontrivial contribution to the photocurrent; 5, $\mathbf{J}_b$ is zero if either the surface states are empty or the surface states are fully occupied. The magnitude of $\mathbf{J}_b$ takes its maximum when the Fermi level is at the Dirac point.

2. optical transitions from surface bands to conduction bands

The derivation of the matrix element and photocurrent is the same as before, except that 1, changing M_v to $M_c \equiv \frac{\hbar^2}{2m_c^*}$; 2, removing the negative sign of the photocurrent since carriers with opposite charges are left behind in surface(conduction) bands. Another thing we would like to emphasize is that in the derivation for photocurrent from surface band to conduction band, we cannot simply exchange index η and ξ. Instead, we need to change B_3 to $-B_3$ besides exchanging η and ξ. Here we keep the assumption that $\xi(\eta)$ denotes the surface(conduction) bands. Therefore, the asymmetric part of the matrix element is

$$|\mathcal{M}|^2_{\eta\xi,a}(s \to c) = -\Delta_{3,M}\sin(2\varphi)\sin(\theta)\sin(\theta_k)\xi\delta_{\xi\eta} \tag{61}$$

where $\Delta_{3,M} = \frac{\sqrt{2}e^2F_3^2A_E^2}{\hbar^2}A_3B_3$

Similarly, we obtain the photocurrent contributed from surface and bulk states as

$$\mathbf{J}_s(E_F, s \to c) = -\frac{e^3F_3^2A_E^2A_3B_3\sin(2\varphi)\sin(\theta)\tau_sS_s^2}{16\sqrt{2}\pi^2\hbar^4A_0M_c}[A_0^2k_c^2 + sgn(E_F)E_F^2]\hat{e}_y \tag{62}$$

$$\mathbf{J}_b(E_F, s \to c) = -\frac{e^3F_3^2A_E^2A_3B_3\sin(2\varphi)\sin(\theta)\tau_cS_s^2}{4\sqrt{2}\pi^2\hbar^4A_0^2\sqrt{M_c}}(E_F + \hbar\omega - E_c)^{\frac{3}{2}}(\frac{4}{15}(\hbar\omega - E_c) - \frac{2}{5}E_F)\hat{e}_y \tag{63}$$

where $\int_a^b x\sqrt{c+x}dx = \frac{2}{5}((c+b)^{\frac{5}{2}} - (c+a)^{\frac{5}{2}}) - \frac{2}{3}((c+b)^{\frac{3}{2}} - (c+a)^{\frac{3}{2}})$ is used during the derivation process. One can easily check that the maximum of function $f(x) = (a+x)^{\frac{3}{2}}(\frac{4}{15}a - \frac{2}{5}x)$ happens when $x = 0$. Thus, the magnitude of $\mathbf{J}_b(E_F, v \to s)$ takes its maximum when $E_F = 0$. It decays as the Fermi level goes away from the Dirac point.

3. Total photocurrent as a function of the Fermi level

In this subsection, we calculate the total photocurrent by combining both contributions to the photocurrent from the above discussions.

$$\mathbf{J}_s(E_F) = -\Pi_{3,j}\tau_s[A_0^2k_c^2(\frac{1}{M_v} + \frac{1}{M_c}) - sgn(E_F)E_F^2(\frac{1}{M_v} - \frac{1}{M_c})]\hat{e}_y \tag{64}$$

, where $\Pi_{3,j} = \Delta_{3,M}\frac{eS_s^2\sin(2\varphi)\sin(\theta)}{32\pi\hbar^2A_0}$. From Eq. 64, we find that if $M_v = M_c$, the total photocurrent contributed from surface bands is independent on the Fermi level. However, the mass of hole is greater than the mass of electron in reality, i.e. $M_v < M_c$. Thus, the magnitude of $\mathbf{J}_s(E_F)$ is greater when $E_F < 0$ and asymmetric about $E_F = 0$.

$$\mathbf{J}_b(E_F) = -4\Pi_{3,j}[\tau_v\frac{(\delta_{E_0} - E_F)^{\frac{3}{2}}(\frac{4}{15}\delta_{E_0} + \frac{2}{5}E_F)}{A_0\sqrt{M_v}} + \tau_c\frac{(E_F + \delta_{E_0})^{\frac{3}{2}}(\frac{4}{15}\delta_{E_0} - \frac{2}{5}E_F)}{A_0\sqrt{M_c}}]\hat{e}_y \tag{65}$$

, where $\delta_{E_0} = E_v + \hbar\omega = \hbar\omega - E_c$. From Eq. 65, we find that the magnitude of total photocurrent conributed from the bulk contribution $\mathbf{J}_b(E_F)$ is maximized at $E_F = 0$ and symmetric about $E_F = 0$ when $M_c = M_v$. $\mathbf{J}_b(E_F)$ becomes asymmetric about $E_F = 0$ when $M_c \neq M_v$.

4. Photocurrent from the Rashba effect

Since there is an external gate voltage applied experimentally to tune the Fermi level, the Rashba effect on the bulk states will play a role here. The Rashba Hamiltonian reads $H_R = \alpha_R(k_y\sigma_x - k_x\sigma_y)$ on the basis $\Psi_{P_3} = (|P_3^+, \uparrow\rangle, |P_3^+, \downarrow\rangle)^T$, where the sign and magnitude of α_R depend on the direction and strength of the external applied electric field. Moreover, α_R is inversely related to the effective mass of bulk states. In this part, we only consider the Rashba effect on the conduction bands, since effective mass of electrons is much smaller than the effective mass of holes.

The modified eigenstates of conduction bands of Ψ_{P_3} can be obtained by solving $H_c = (E_c + M_ck^2)\sigma_0 + \alpha_R(k_y\sigma_x - k_x\sigma_y)$. Denote $\alpha_R = s_\alpha|\alpha_R|$ with $s_\alpha = \pm 1$. The eigenstate reads $\phi_{R,c,\mathbf{k},\eta} = \frac{1}{\sqrt{2}}(i\eta s_\alpha e^{-i\theta_k}, 1)^T$ with eigenenergy $E_\eta = E_c + M_ck^2 + \eta|\alpha_Rk|$ and $\eta = \pm 1$. Similarly, one can obtain the asymmetric part of matrix element as

$$|\mathcal{M}|^2_{R,\eta\xi,a}(s \to c) = -\Delta_{3,M}\sin(2\varphi)\sin(\theta)\sin(\theta_k)\xi\delta_{\xi,\eta s_\alpha} \tag{66}$$

The velocity of bulk states now changes to $\mathbf{v}_{\mathbf{k},\eta} = (2M_c|k| + \eta|\alpha_R|)\cos(\theta_k)\hat{e}_x + (2M_c|k|\sin(\theta_k) + \eta|\alpha_R|)\hat{e}_y$. Similarly, the photocurrent for optical transitions from surface bands to conduction bands can be expressed as

$$\mathbf{J}_{R,s}(E_F, s \to c) = -\Pi_{3,j}\tau_s \frac{1}{M_c}[A_0^2 k_c^2 + sgn(E_F)E_F^2]\hat{e}_y \tag{67}$$

$$\mathbf{J}_{R,b}(E_F, s \to c) = -\Pi_{3,j}\tau_c[\frac{1}{A_0\sqrt{M_c}}(E_F + \hbar\omega - E_c + \frac{\alpha_R^2}{4M_c})^{\frac{3}{2}}(\frac{4}{15}(\hbar\omega - E_c + \frac{\alpha_R^2}{4M_c}) - \frac{2}{5}E_F)$$
$$+\frac{\alpha_R}{4A_0M_c}(A_0^2k_c^2 - sgn(E_F)E_F^2)]\hat{e}_y \tag{68}$$

From the equations above, we find that the Rashba splitting on the conduction bands does not affect the photocurrent contributed by the surface states. However, it indeed influences the photocurrent contributed from the conduction bands with $J_{R,b,extra} \sim -\alpha_R(A_0^2k_c^2 - sgn(E_F)E_F^2)\hat{e}_y$ and makes the photocurrent more asymmetric about $E_F = 0$.

Bulk states: $|P_4^-, \sigma\rangle$

The derivation of the transition matrix element and photocurrent for $|P_4^-, \sigma\rangle$ states is the same as that for states $|P_3^+, \sigma\rangle$, except that the nonzero interaction terms come from coupling $\langle P_+^1, \sigma|\hat{\mathbf{p}}|P_4^-\rangle$. The above derivation can be applied here directly by just replacing A_3, B_3 with A_4, B_4.

Bulk states: $|P_5^-, \pm\frac{3}{2}\rangle$ and $|P_6^+, \pm\frac{3}{2}\rangle$

In this subsection, we will first consider the contribution to photocurrent for optical transitions from valence bands to surface bands for $|P_5^-, \pm\frac{3}{2}\rangle$. Recall that surface states $\phi_{s,\mathbf{k},\xi} = \frac{1}{\sqrt{2}}(i\xi e^{-i\theta_k}, 1)^T$ and bulk states $\phi_{b,\mathbf{k},\eta} = \frac{1}{\sqrt{2}}(i\eta e^{-i\theta_k}, 1)^T$, i.e. $d_\xi = \frac{1}{\sqrt{2}}f(z)$, $d_\eta = g(z)$, $c_{\xi,|P_1^+,\uparrow\rangle} = \frac{1}{\sqrt{2}}i\xi e^{-i\theta_k}$, $c_{\xi,|P_1^+,\downarrow\rangle} = \frac{1}{\sqrt{2}}$, $c_{\eta,|P_5^-,\frac{3}{2}\rangle} = \frac{1}{\sqrt{2}}i\eta e^{-i\theta_k}$ and $c_{\eta,|P_5^-,-\frac{3}{2}\rangle} = \frac{1}{\sqrt{2}}$. Thus, we have

$$\mathcal{D}_{\xi\eta}(v \to s) = -\frac{ieF_3}{2\hbar}[((1-\xi\eta)B_5 + (\eta e^{-i\theta_k} + \xi e^{i\theta_k})A_5)/\sqrt{2}\hat{e}_x + ((1+\xi\eta)B_5 + (\eta e^{-i\theta_k} - \xi e^{i\theta_k})A_5)/\sqrt{2}\hat{e}_y] \tag{69}$$

where $F_5 = \sum_z \frac{1}{\sqrt{2}}f^*(z)g(z)$, which is independent of the momentum by using small k approximation.

Recall that $A_x = -(1 - i\cos(2\varphi))\cos(\theta)A_E$, $A_y = -i\sin(2\varphi)A_E$ and $A_z = -(1 - i\cos(2\varphi))\sin(\theta)A_E$. Now, we are ready to calculate the matrix element $|\mathcal{M}|^2_{\xi\eta}$.

$$|\mathcal{M}|^2_{\xi\eta}|^2(v \to s) = |-(1 - i\cos(2\varphi))\cos(\theta)A_E\mathcal{D}^x_{\xi\eta} - i\sin(2\varphi)A_E\mathcal{D}^y_{\xi\eta}|^2 \tag{70}$$
$$= \frac{e^2F_3^2A_E^2}{8\hbar^2}[\cos^2(\theta)(1-\xi\eta)^2B_5^2(1+\cos^2(2\varphi)) + (1+\eta\xi)^2B_5^2\sin^2(2\varphi) + 2\cos^2(\theta)(1+\eta\xi\cos(2\theta_k))A_5^2(1+\cos^2(2\varphi))$$
$$+2(1-\eta\xi\cos(2\theta_k))A_5^2\sin^2(2\varphi) + 2A_5^2\eta\xi\sin(2\theta_k)\sin(2\varphi)] - \frac{e^2F_3^2A_E^2}{\hbar^2}\eta\sin(4\varphi)\cos(\theta)A_5B_5\cos(\theta_k) \tag{71}$$

, where $(1-\eta\xi)(1+\eta\xi) = 0$ is used. Therefore, the asymmetric part of matrix element is

$$|\mathcal{M}|^2_{5,\xi\eta,a}(v \to s) = -\frac{e^2F_3^2A_E^2}{\hbar^2}\eta\sin(4\varphi)\cos(\theta)A_5B_5\cos(\theta_k) \tag{72}$$

Though the asymmetric part of the transition matrix element exists, there is no net photocurrent contributed from $|P_5^- \pm \frac{3}{2}\rangle$ bulk states. The reason is that for a surface state with index ξ, both degenerate bulk eigenstates with index $\eta = \pm 1$ will contribute to the matrix element with opposite signs, leading to zero net photocurrent. Similarly, optical transitions from surface bands to conduction bands also result in zero net photocurrent. The conclusion of zero net photocurrent can be applied to optical transitions between surface bands and $|P_6^+, \pm\frac{3}{2}\rangle$ states.

This conclusion verifies the result of circularly polarized galvanic effect from the symmetry principle[7], where the photocurrent constrained by the three-fold rotational symmetry $C_3(z)$ and mirror symmetry M_x in Bi-chalcogenides is written as

$$J_{\text{CPGE}} = i\gamma[(A_zA_x^* - A_xA_z^*)\hat{\mathbf{x}} - (A_yA_z^* - A_zA_y^*)\hat{\mathbf{y}}] \tag{73}$$

where γ is a material-dependent parameter. Thus, one needs A_z term to play an essential role for nonzero photocurrent. From the above calculation, we know that $\mathcal{D}_z = 0$ for optical transitions between $P_5^-(P_6^+)$ states and surface states. Since the matrix element couples $\mathcal{D}_z$ and A_z, A_z will not be included in the calculation of photocurrent. Thus, there is no photocurrent induced by optical transitions with $P_5^-(P_6^+)$ states involved.

SUPPLEMENTARY NOTE 4 – EFFECT OF THE RELAXATION TIME OF EXCITED CARRIERS ON THE HDPC

In the main text, we have presented numerical results of the HDPC with the assumption that the relaxation times of excited carriers in different bands are the same. This is a very rough estimation which may deviate from the real condition. There are several time-resolved experiments studying the relaxation process of light excited carriers in topological insulators [8–13]. Though it is agreed that the electron-phonon scattering is the dominant cause of the momentum relaxation, the time scale of the relaxation process varies between experiments. Specifically, the momentum relaxation times of the excited surface carriers vary from 0.3 ps to 6 ps. Two stages of decay process reported by Ref [11] add more complexity to the relaxation of the photocurrent. In addition, some experiments reported that the excited carriers in the bulk bands have a slightly longer relaxation time compared to the surface carriers [10, 12]. The energy dependence of the relaxation process is also not straightforward. Within the same band, it is demonstrated that higher energy state decays faster due to the intraband scattering, however, a different bulk band at higher energy may not have a faster relaxation compared to a lower energy bulk band since this involves a typically slower process- interband scattering. Therefore, we cannot simply assume that higher energy band has a smaller relaxation time.

Though the relaxation time for each band is still under debate, we can still use different relaxation times for each band to explore the influence of different relaxation times on the HDPC. The contributions to the photocurrent from the conduction band and the valence band are also separated and compared in this section. The parameters for the numerical calculation remain the same as the main text except for relaxation times for each band, τ_s, τ_c and τ_v.

1, Relaxation time of surface states is different from that of the bulk states

In this subsection, we assume that the carriers in the conduction bands and valence bands have the same relaxation time, i.e. $\tau_c = \tau_v$. We gradually change the relaxation time for the carriers in the surface states. Supplementary Figure 5 shows the computational results for the cases with $\tau_s = 0.5\tau_v$, τ_v and $2\tau_v$, respectively. We find that as the relaxation time for carriers of surface states increases, the photocurrent contributed by the surface carriers increases. However, different relaxation times only change the total photocurrent quantitatively while all the essential qualitative features, such as the peak at the Dirac point and the asymmetry between the electron and hole doping regimes, remain the same. More quantitatively, by comparing the lineshape of the calculated photocurrents with that in experiments, we conclude that a shorter surface relaxation time (Supplementary Figure 7(a)) gives rise to a better match with the experimental observations. This finding is consistent with the time resolved studies of carriers relaxation in topological insulators. [10, 12]

2, Relaxation time of the conduction band is different from that of the valence band

In this subsection, we assume that the carriers in the surface states and valence bands have the same relaxation time, i.e. $\tau_s = \tau_v$. We gradually change the relaxation time for the carriers in the conduction band. The relaxation time of carriers in conduction bands, as shown in Supplementary Fig. 6 is $\tau_c = 0.5\tau_v$, τ_v and $2\tau_v$, respectively. We find that as the relaxation time for carriers of the conduction band increases, the photocurrent contributed by the conduction band carriers increases and also becomes more asymmetric around the Dirac point. Moreover, the tunability of the photocurrent with the chemical potential increases as the relaxation time for the conduction band increases.

In reality, we could expect that the relaxation time of the conduction band, valence band, surface states are all different. This is why we are not seeing an exactly same HDPC as the experimental observation in the simplified numerical calculation taking the same momentum relaxation time for all bands. However, when the numerical calculation takes into account the difference of the relaxation time between the bands, like Supplementary Fig. 5 and Supplementary Fig. 6, the HDPC may be closer to what we observe in the experiment.

SUPPLEMENTARY NOTE 5 – PHOTOCURRENT FROM OPTICAL TRANSITIONS BETWEEN TWO DIRAC-CONE SURFACE STATES

Since there appears a second Dirac-cone surface states above the first Dirac cone, we need to consider the contribution to the photocurrent between the two Dirac-cone surface states. The Hamiltonian of the second surface state

can be written as

$$H_{s2} = A_{s2}(k_y\sigma_x - k_x\sigma_y) \tag{74}$$

Similarly, one can solve for the two eigenstates of the surface states, expressed as $\phi_{s2,\mathbf{k},\eta} = \frac{1}{\sqrt{2}}(i\eta e^{-i\theta_k}, 1)^T$ with eigenenergy $E_{s,2,k,\eta} = A_{s2}\eta|k|$, where $\eta = \pm 1$ labels the upper/lower Dirac cone.

If velocities of two Dirac-cone surface states are the same, i.e., $A_0 = A_{s2}$ and the photon energy matches the energy difference of two surface states, we have the photocurrent

$$\mathbf{J}(s \to s_2) = -\frac{2\pi e}{\hbar}\sum_{\mathbf{k},\langle\xi,\eta\rangle}(\tau_{s2}\mathbf{v}_{\mathbf{k},\eta} - \tau_s\mathbf{v}_{\mathbf{k},\xi})|\mathcal{M}|^2_{\eta\xi,a}(f^0_{\mathbf{k},\xi} - f^0_{\mathbf{k},\eta})\delta(E_{\mathbf{k},\eta} - E_{\mathbf{k},\xi} - \hbar\omega) \tag{75}$$

where τ_s (τ_{s2}) is the relaxation time for carriers of the (first) second Dirac-cone surface states, $\xi(\eta)$ denotes the first and second Dirac-cone surface states. If the velocities of two Dirac cone surface states are different, i.e. $A_0 \neq A_{s,2}$, only states on a specific ring in the momentum space can match the photon energy we use. For such a case, the contribution to the net photocurrent would be much smaller than that for the previous discussed cases. Therefore, we only consider the situation with $A_0 = A_{s2}$. The difference between τ_s and τ_{s2} plays an essential role for the nonzero photocurrent for such situations[14].

Recall that the first Dirac-cone surface states $\phi_{s,\mathbf{k},\xi} = \frac{1}{\sqrt{2}}(i\xi e^{-i\theta_k}, 1)^T$ and the second Dirac-cone surface states $\phi_{s2,\mathbf{k},\eta} = \frac{1}{\sqrt{2}}(i\eta e^{-i\theta_k}, 1)^T$, i.e. $d_\xi = \frac{1}{\sqrt{2}}f(z)$, $d_\eta = \frac{1}{\sqrt{2}}f_2(z)$, $c_{\xi,|P_1^+,\uparrow\rangle} = \frac{1}{\sqrt{2}}i\xi e^{-i\theta_k}$,$c_{\xi,|P_2^-,\uparrow\rangle} = \frac{1}{\sqrt{2}}i\xi e^{-i\theta_k}$, $c_{\xi,|P_1^+,\downarrow\rangle} = \frac{1}{\sqrt{2}}$, $c_{\xi,|P_2^-,\downarrow\rangle} = \frac{1}{\sqrt{2}}$, $c_{\eta,|P_3^+,\uparrow\rangle} = \frac{1}{\sqrt{2}}i\eta e^{-i\theta_k}$, $c_{\eta,|P_4^-,\uparrow\rangle} = \frac{1}{\sqrt{2}}i\eta e^{-i\theta_k}$, $c_{\eta,|P_3^+,\downarrow\rangle} = \frac{1}{\sqrt{2}}$ and $c_{\eta,|P_4^-,\downarrow\rangle} = \frac{1}{\sqrt{2}}$. Here we assume the second Dirac-cone surface states originate from the $|P_3^+,\sigma\rangle$ and $|P_4^-,\sigma\rangle$ states. Furthermore, we can solve for $\mathcal{D}_{\eta\xi}$ as $\mathcal{D}^z_{\eta\xi}(s \to s_2) = \frac{e}{m}\sum_{z,n,n'} d^*_\eta(\mathbf{k},z)d_\xi(\mathbf{k},z)c^*_{\eta,n'}(\mathbf{k})c_{\xi,n}(\mathbf{k})P^z_{nn'} = \frac{e}{m}\sum_z \frac{1}{\sqrt{2}}f^*_2(z)\frac{1}{\sqrt{2}}f(z)(c^*_{\eta,|P_3^+,\uparrow\rangle}c_{\xi,|P_2^-,\uparrow\rangle}(-i\frac{m}{\hbar}B_3) + c^*_{\eta,|P_3^+,\downarrow\rangle}c_{\xi,|P_2^-,\downarrow\rangle}(-i\frac{m}{\hbar}B_3) + c^*_{\eta,|P_4^-,\uparrow\rangle}c_{\xi,|P_1^+,\uparrow\rangle}(-i\frac{m}{\hbar}B_4) + c^*_{\eta,|P_4^-,\downarrow\rangle}c_{\xi,|P_1^+,\downarrow\rangle}(-i\frac{m}{\hbar}B_4)) = -\frac{ieF_{34}}{2\hbar}(\eta\xi + 1)(B_3 + B_4)$ where $F_{34} = \sum_z \frac{1}{2}f^*_2(z)f(z)$, which is independent of momentum by using small k approximation. Similarly, $\mathcal{D}^+_{\eta\xi}(s \to s_2) = \frac{e}{m}\sum_{z,n,n'} d^*_\eta(\mathbf{k},z)d_\xi(\mathbf{k},z)c^*_{\eta,n'}(\mathbf{k})c_{\xi,n}(\mathbf{k})P^+_{nn'} = \frac{e}{m}\sum_z \frac{1}{\sqrt{2}}f^*_2(z)\frac{1}{\sqrt{2}}f(z)(c^*_{\eta,|P_3^+,\uparrow\rangle}c_{\xi,|P_2^-,\downarrow\rangle}i\frac{m}{\hbar}A_3 + c^*_{\eta,|P_4^-,\uparrow\rangle}c_{\xi,|P_1^+,\downarrow\rangle}i\frac{m}{\hbar}A_4) = \frac{eF_{34}}{2\hbar}\xi e^{i\theta_k}(A_3 + A_4)$ and $\mathcal{D}^-_{\eta\xi}(s \to s_2) = \frac{e}{m}\sum_{z,n,n'} d^*_\eta(\mathbf{k},z)d_\xi(\mathbf{k},z)c^*_{\eta,n'}(\mathbf{k})c_{\xi,n}(\mathbf{k})P^+_{nn'} = \frac{eF_{34}}{2\hbar}\eta e^{-i\theta_k}(A_3 + A_4)$

Thus, we have

$$\mathcal{D}_{\eta\xi}(s \to s_2) = -\frac{ieF_{34}}{2\hbar}[(\eta\xi + 1)(B_3 + B_4)\hat{e}_z + (i\eta e^{-i\theta_k} + i\xi e^{i\theta_k})(A_3 + A_4)/\sqrt{2}\hat{e}_x - (\eta e^{-i\theta_k} - \xi e^{i\theta_k})(A_3 + A_4)/\sqrt{2}\hat{e}_y]$$

where $\mathcal{D}^+_{\xi\eta}(v \to s) = \frac{1}{\sqrt{2}}(\mathcal{D}^x_{\xi\eta} + i\mathcal{D}^y_{\xi\eta})$ and $\mathcal{D}^-_{\xi\eta}(v \to s) = \frac{1}{\sqrt{2}}(\mathcal{D}^x_{\xi\eta} - i\mathcal{D}^y_{\xi\eta})$ are used. The asymmetric part of matrix element, $|\mathcal{M}_{\eta\xi}|^2_a$, can be further calculated and expressed as

$$|\mathcal{M}_{\eta\xi}|^2_a(s \to s_2) = -\Delta_{s2,M}\sin(2\varphi)\sin(\theta)\sin(\theta_k)\xi\delta_{\xi\eta} \tag{76}$$

where $\Delta_{s2,M} = \frac{\sqrt{2}e^2F^2_{34}A^2_E}{\hbar^2}(A_3 + A_4)(B_3 + B_4)$ in unit of eV and $\xi = \pm 1$ ($\eta = \pm 1$) indicates upper/lower Dirac cone with left-handed/right-handed spin texture for the first Dirac-cone surface states (the second Dirac-cone surface states).

Once the photon energy we use matches the energy difference of the two Dirac-cone surface states, We have

$$\begin{aligned}
\mathbf{J}(s \to s_2) &= -\frac{2\sqrt{2}\pi e^3F^2_{34}A^2_EA_0(A_3 + A_4)(B_3 + B_4)\sin(2\varphi)\sin(\theta)}{\hbar^4}(\tau_{s2} - \tau_s)\hat{e}_y\sum_{\mathbf{k},\langle\xi,\eta\rangle}\delta_{\xi\eta}\sin^2(\theta_k)(f^0_{\mathbf{k},\eta} - f^0_{\mathbf{k},\xi})\delta(E_{\mathbf{k},\xi} - E_{\mathbf{k},\eta} - \hbar\omega) \\
&= \frac{2\sqrt{2}\pi e^3F^2_{34}A^2_EA_0(A_3 + A_4)(B_3 + B_4)\sin(2\varphi)\sin(\theta)}{\hbar^4}(\tau_s - \tau_{s2})\hat{e}_y\frac{S_s}{4\pi^2} \times \\
&\quad [\Theta(-E_F)\int_{-k_c}^{k_F}|k|dk\int_0^{2\pi}\sin^2(\theta_k)d\theta_k + \Theta(E_F)(\int_0^{k_F}kdk\int_0^{2\pi}\sin^2(\theta_k)d\theta_k + \int_{-k_c}^{0}|k|dk\int_0^{2\pi}\sin^2(\theta_k)d\theta_k] \\
&= \frac{e^3F^2_{34}A^2_E(A_3 + A_4)(B_3 + B_4)S_s\sin(2\varphi)\sin(\theta)}{2\sqrt{2}A_0\hbar^4}(\tau_s - \tau_{s2})[A^2_0k^2_c + sgn(E_F)E^2_F]\hat{e}_y
\end{aligned} \tag{77}$$

where $\mathbf{v}_{s,\mathbf{k},\xi} = \frac{A_0}{\hbar}\xi\cos(\theta_k)\hat{e}_x + \frac{A_0}{\hbar}\xi\sin(\theta_k)\hat{e}_y$, k_F is the Fermi momentum and k_c is the cut-off momentum. We still assume that the condition $E_{\mathbf{k},\xi} - E_{\mathbf{k},\eta} - \hbar\omega = 0$ can be always fulfilled and the temperature is $T = 0$K during the derivation process.

We find that optical transitions between two Dirac-cone surface states could also contribute to the photocurrent, which is proportional to the difference of relaxation times for carriers of the two surface states. From the derivation, we also find that as the Fermi level moves up from valence bands to conduction bands, the magnitude of photocurrent contributed by the optical transitions between the two Dirac-cone surface states increases. However, our experimental observation of the gate dependent photocurrent does not match with the photocurrent dependence on the Fermi level here. We learn that the helicity dependent photocurrent contributed by the optical transitions between the first and second surface states is not the major effect in our observation.

SUPPLEMENTARY REFERENCES

[1] J. W. McIver, D. Hsieh, H. Steinberg, P. Jarillo-Herrero, and N. Gedik, Nat. Nanotech. **7**, 96 (2012).
[2] L. Braun, G. Mussler, A. Hruban, M. Konczykowski, M. Wolf, T. Schumann, M. Münzenberg, L. Perfetti, and T. Kampfrath, Nature Communications **7**, 13259 (2016).
[3] D. A. Bas, R. A. Muniz, S. Babakiray, D. Lederman, J. Sipe, and A. D. Bristow, Optics express **24**, 23583 (2016).
[4] C. Kastl, T. Guan, X. He, K. Wu, Y. Li, and A. Holleitner, Applied Physics Letters **101**, 251110 (2012).
[5] C.-X. Liu, X.-L. Qi, H. Zhang, X. Dai, Z. Fang, and S.-C. Zhang, Physical Review B **82**, 045122 (2010).
[6] H. Zhang, C.-X. Liu, X.-L. Qi, X. Dai, Z. Fang, and S.-C. Zhang, Nature physics **5**, 438 (2009).
[7] A. Junck, *Theory of Photocurrents in Topological Insulators*, Ph.D. thesis, im Fachbereich Physik der Freien Universitat Berlin eingereichte (2015).
[8] J. A. Sobota, S. Yang, J. G. Analytis, Y. Chen, I. R. Fisher, P. S. Kirchmann, and Z.-X. Shen, Physical Review Letters **108**, 117403 (2012).
[9] C. Kastl, C. Karnetzky, H. Karl, and A. W. Holleitner, Nature Communications **6**, 6617 (2015).
[10] D. Hsieh, F. Mahmood, J. McIver, D. Gardner, Y. Lee, and N. Gedik, Physical Review Letters **107**, 077401 (2011).
[11] Y. Wang, D. Hsieh, E. Sie, H. Steinberg, D. Gardner, Y. Lee, P. Jarillo-Herrero, and N. Gedik, Physical Review Letters **109**, 127401 (2012).
[12] N. P. Butch, K. Kirshenbaum, P. Syers, A. B. Sushkov, G. S. Jenkins, H. D. Drew, and J. Paglione, Physical Review B **81**, 241301 (2010).
[13] J. Sánchez-Barriga, E. Golias, A. Varykhalov, J. Braun, L. Yashina, R. Schumann, J. Minár, H. Ebert, O. Kornilov, and O. Rader, Physical Review B **93**, 155426 (2016).
[14] S. Ganichev, V. BelÕkov, P. Schneider, E. Ivchenko, S. Tarasenko, W. Wegscheider, D. Weiss, D. Schuh, E. Beregulin, and W. Prettl, Physical Review B **68**, 035319 (2003).
